# Advances in field-effect biosensors towards point-of-use

**DOI:** 10.1088/1361-6528/acf3f0

**Published:** 2023-09-25

**Authors:** Sihan Chen, Rashid Bashir

**Affiliations:** 1 Holonyak Micro and Nanotechnology Laboratory, The Grainger College of Engineering, University of Illinois Urbana-Champaign, Urbana, IL 61801, United States of America; 2 Department of Bioengineering, The Grainger College of Engineering, University of Illinois Urbana-Champaign, Urbana, IL 61801, United States of America; 3 Department of Biomedical and Translational Sciences, Carle Illinois College of Medicine, University of Illinois Urbana-Champaign, Urbana, IL 61801, United States of America

**Keywords:** field-effect transistor, FET, biosensor, electrochemical, point-of-care, diagnostics

## Abstract

The future of medical diagnostics calls for portable biosensors at the point of care, aiming to improve healthcare by reducing costs, improving access, and increasing quality—what is called the ‘triple aim’. Developing point-of-care sensors that provide high sensitivity, detect multiple analytes, and provide real time measurements can expand access to medical diagnostics for all. Field-effect transistor (FET)-based biosensors have several advantages, including ultrahigh sensitivity, label-free and amplification-free detection, reduced cost and complexity, portability, and large-scale multiplexing. They can also be integrated into wearable or implantable devices and provide continuous, real-time monitoring of analytes *in vivo*, enabling early detection of biomarkers for disease diagnosis and management. This review analyzes advances in the sensitivity, parallelization, and reusability of FET biosensors, benchmarks the limit of detection of the state of the art, and discusses the challenges and opportunities of FET biosensors for future healthcare applications.

## 1. Introduction

Biosensors are devices that detect the presence and measure the quantity or concentration of biological analytes in samples, with broad and critical applications in healthcare, food safety, and environmental monitoring [1, 2]. However, traditional biosensors in healthcare often require specialized technical staff and expensive equipment, leading to long wait times for test results and limited accessibility in resource-limited areas. To address these challenges, point-of-care (POC) biosensors are being developed that are easy to use, portable, and rapid. POC improves healthcare by reducing costs, increasing efficiency, and minimizing the time between diagnosis and treatment.

Electronic biosensors offer several advantages in POC applications. First, they can detect biomolecules without labeling or amplification, simplifying the assay and reducing both time and cost. Second, they can be made small, portable, and mass-produced at a low cost. Third, they can be integrated with signal processing and wireless data transmission units on a single chip, enabling seamless integration with electronic health records and telemedicine platforms for remote monitoring and decision-making. Fourth, they can provide continuous, real-time monitoring of analytes, allowing early detection of changes in biomarker levels indicative of disease progression or response to treatment.

Field-effect transistor (FET)-based biosensors, also known as FET biosensors or bioFETs, are widely used in electronic biosensing. Compared to impedance-based and electrochemical biosensors, bioFETs have the advantage of significantly lower detection limits [3].

In this review, our focus is on the advances in sensitivity, parallelization, and reusability of bioFETs. We begin by discussing the trends in field-effect biosensing, and briefly review the basic device structure and working principles of bioFETs. We then elaborate on the advantages and limitations of nanobioFETs. Next, we benchmark the limit of detection of state-of-the-art bioFETs for nucleic acids, proteins, small biomolecules, and ions. From there, we take a fundamental and unified perspective to analyze key innovations that enhance the sensitivity and limit of detection of bioFETs, irrespective of the analytes. We also review various strategies to overcome the Debye limit at physiological ionic strength. Afterwards, we elucidate the benefits of parallelization with two examples of million-bioFET arrays, followed by discussions on advances in reusable biosensors. Finally, we identify the challenges and opportunities to commercialize bioFETs for future healthcare applications. This review does not address the topic of cell sensing using FET biosensors. Previous reviews have already covered the detection of cells (bacteria, tumor cells, etc) [4–6], as well as the interfacing of cells (particularly neurons) [7] with bioFETs, and these topics are not covered here in this review.

**Figure 1.**
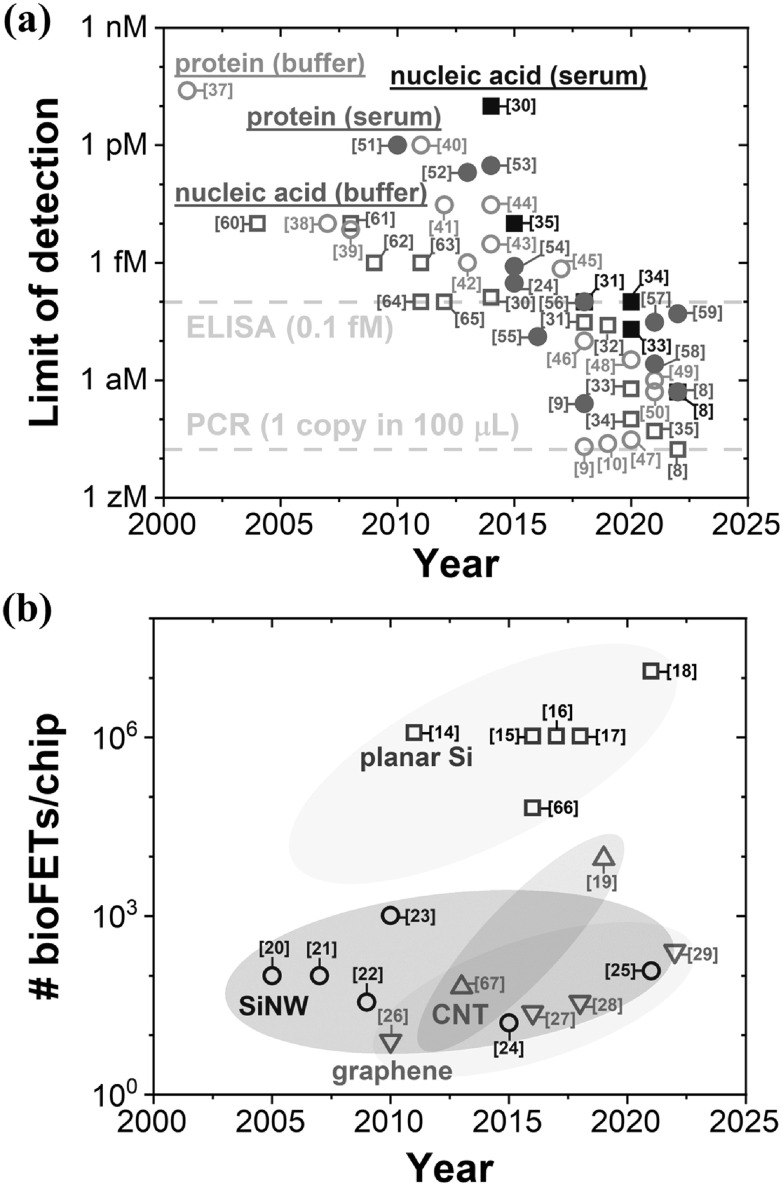
Trends in field-effect biosensing. (a) Limit of detection of nucleic acid and protein in buffer and in serum versus year for bioFETs [8–10, 24, 30–65]. As a benchmark, PCR and ELISA technologies could detect nucleic acid and protein down to 1 copy per 100 *μ*l [11] and ∼0.1 fM (∼60 million proteins with a molecular weight of 50 kDa per *μ*l) [12, 13], respectively. (b) Number of bioFETs per chip versus year for four different channel materials: planar silicon [14–18, 66], silicon nanowire (SiNW) [20–25], carbon nanotube (CNT) [19, 67], and graphene [26–29]. For each data point, the reference is shown in brackets. This figure contains key developments in sensitivity and parallelization and is by no means exhaustive.

Figure 1(a) shows the trend in the limit of detection (LoD) of field-effect biosensing, using the detection of nucleic acids and proteins as an example. Over the past two decades, the LoD of nucleic acids (DNA or RNA) has improved from ∼10 fM in buffer and >10 pM in serum to 17 zM in buffer and 500 zM in serum [8]. In the case of proteins, the LoD has improved from >1 pM in buffer and serum to ∼20 zM in buffer [9, 10] and 250 zM in serum [9]. For comparison, the gold standard test in nucleic acid sensing—polymerase chain reaction (PCR)—can detect nucleic acid down to 1 copy per 100 *μ*l [11], i.e. 17 zM. The gold standard in protein detection—enzyme-linked immunosorbent assay (ELISA)—can detect proteins down to ∼0.1 fM [12, 13]. Therefore, state-of-the-art bioFETs are now as sensitive as PCR in nucleic acid detection and even more sensitive than ELISA in protein detection.

We caution that the various measurements reported in figure 1(a) are for different detection time—the time elapsed from the introduction of the sample to the biosensor until the detection signal reaches a predefined threshold. Achieving a lower LoD with a longer detection time does not necessarily indicate better performance than achieving a higher LoD with a shorter detection time. This is because a longer detection time can lead to a lower LoD when mass transport limits the LoD, a topic we will address in detail later.

Figure 1(b) shows the number of bioFETs per chip for various channel materials over the years. Planar silicon-based bioFETs have achieved an impressive integration of over a million bioFETs on a single chip [14–18]. Carbon nanotube (CNT)-based bioFETs have also shown a significant progress in packing density, reaching ∼10 000 bioFETs per chip [19]. On the other hand, the number of bioFETs per chip for silicon nanowire (SiNW) has been limited to 100–1000 over the past two decades [20–25]. At the same time, the packing density of graphene-based bioFETs [26–29] has increased from ∼10 bioFETs per chip to 256 bioFETs per chip.

In general, the degree of parallelization of bioFETs made from a particular channel material is higher if the integrated circuit (IC) technology of that material is more mature. As the device stability, repeatability, reproducibility, and device-to-device variation of nanomaterials such as silicon nanowires, carbon nanotubes, and graphene continue to improve, their degree of parallelization is expected to eventually match that of planar silicon.

## 2. Device structure and working principles

**Figure 2.**
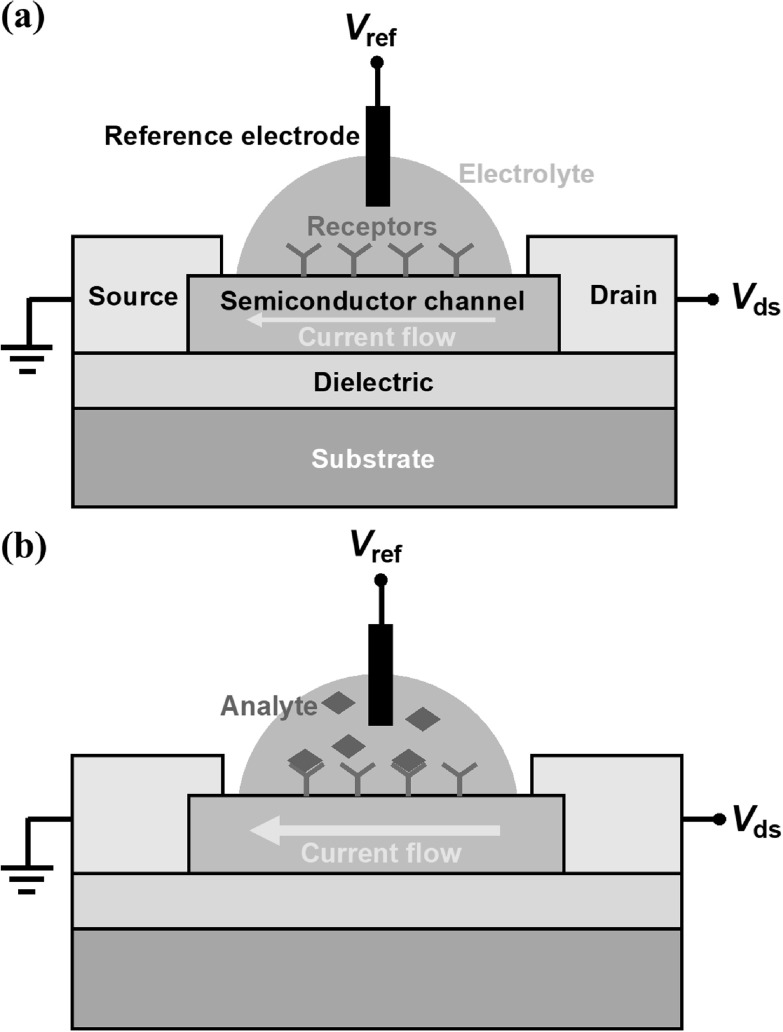
Basic device structure and working principle. (a) Schematic of an electrolyte-gated bioFET. The channel surface is functionalized with bio-receptors. Drain-source bias *V*_ds_ is applied and drain current is measured. A reference electrode applies a bias *V*_ref_ to gate the semiconductor channel via the electrolyte. (b) Schematic of an electrolyte-gated bioFET for the detection of the analytes. Analytes bind to the receptors immobilized on the channel surface and changes the surface potential of the channel, which in turn changes the current of the FET.

Figure 2 illustrates the basic device structure and working principle of a bioFET. As shown in figure 2(a), the basic device structure of a bioFET consists of a source electrode, a drain electrode, a semiconductor channel that connects the source and drain, and a reference electrode that couples to the semiconductor channel via the electrolyte. Receptors that selectively capture the analyte are immobilized on the semiconductor channel. A dielectric layer insulates the bioFET from the substrate.

The bioFET operates on the principle that the binding of the target analyte to the receptors on the channel surface alters the surface potential and consequently the channel conductance. Specifically, a drain-source bias *V*_ds_ drives the current flow in the semiconductor channel, which is controlled by the surface potential of the channel. When a reference electrode applies a bias *V*_ref_ in the electrolyte, it generates a potential drop between the channel surface and some distance into the electrolyte. When the target analyte binds to the receptors on the channel surface, the surface potential of the channel changes, which in turn alters the channel conductance (figure 2(b)).

**Figure 3.**
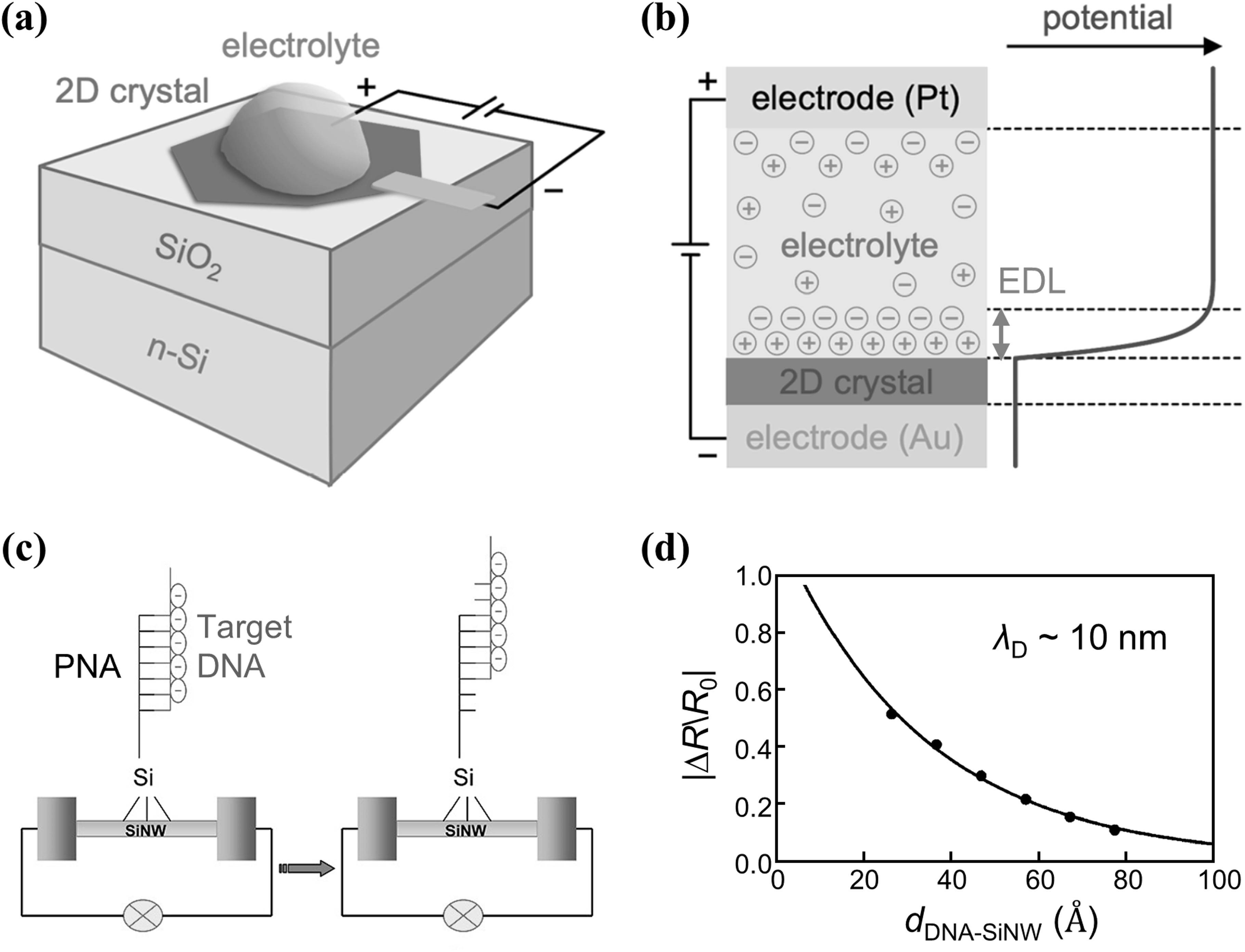
Debye screening limited field-effect sensing. (a) Schematic of electrolyte top-gating. The voltage is applied between the ionically conducting electrolyte and 2D crystal. (b) Potential profile for electrolyte gating. Most of the voltage drop occurs within the electrical double-layer (EDL) at the 2D crystal/electrolyte interface. (c) Schematic of silicon nanowire FET biosensors with different hybridization sites of target DNA to PNA. (d) Plots of the relative change in resistance |Δ*R*/*R*_0_| versus calculated distance of DNA strands to the silicon nanowires *d*_DNA-SiNW_. The filled circles are the experimental data, and the solid line is the least-squares fit to the data. The Debye length *λ*_D_ was ∼10 nm. Figures reproduced with permission from: (a)–(b) [68], © 2021 American Chemical Society (ACS); (c)–(d) Reprinted with permission from [69]. Copyright (2008) American Chemical Society.

Ideally, the charges of the analyte captured by receptors on the channel surface are balanced by changes in the charges of the charge carriers in the semiconductor channel. However, we must consider the role of ions in the electrolyte in maintaining charge balance. As illustrated in figures 3(a) and (b), when a bias is applied between the electrolyte and a 2D crystal, ions in the electrolyte re-arrange and build up near the charged surface to form an ‘electric double layer (EDL)’. Most of the voltage drop occurs within the EDL at the 2D crystal/electrolyte interface [68]. This EDL limits the performance of any bioFET as the analyte must penetrate it to be detected by the semiconductor channel. The thickness of EDL can be estimated by Debye length, which is the distance over which the electrostatic potential drops by 1/*e*. In figures 3(c) and (d), experimental results show that the field-effect response of a SiNW sensor decreases as the DNA moves away from the SiNW surface [69], demonstrating the impact of charge screening on the sensing performance of bioFETs.

Before FET sensors were used to detect biomolecules, they have been applied to measure the pH of solutions in commercial products for decades. In pH sensing, the sensor surface presents hydroxyl or amino groups [70], which act as receptors for hydrogen ions by undergoing protonation and deprotonation reactions that modulate the charge density of the sensor surface. The isoelectric point (pI) marks the pH where the sensor surface has no net charge. At higher pH (>pI), the sensor surface has a more negative charge, as is the case for SiO_2_ or Si_3_N_4_ dielectrics, whereas at lower pH (<pI), the sensor surface has a more positive charge. Minute pH changes occur in many biological processes due to proton release or uptake by the biochemical reactions involved [71]. Therefore, pH sensing is useful for monitoring numerous biochemical processes, as discussed in [5], and these FET based pH sensors are commonly used as commercial pH meters.

## 3. Advantages and limitations of nanobioFETs

**Figure 4.**
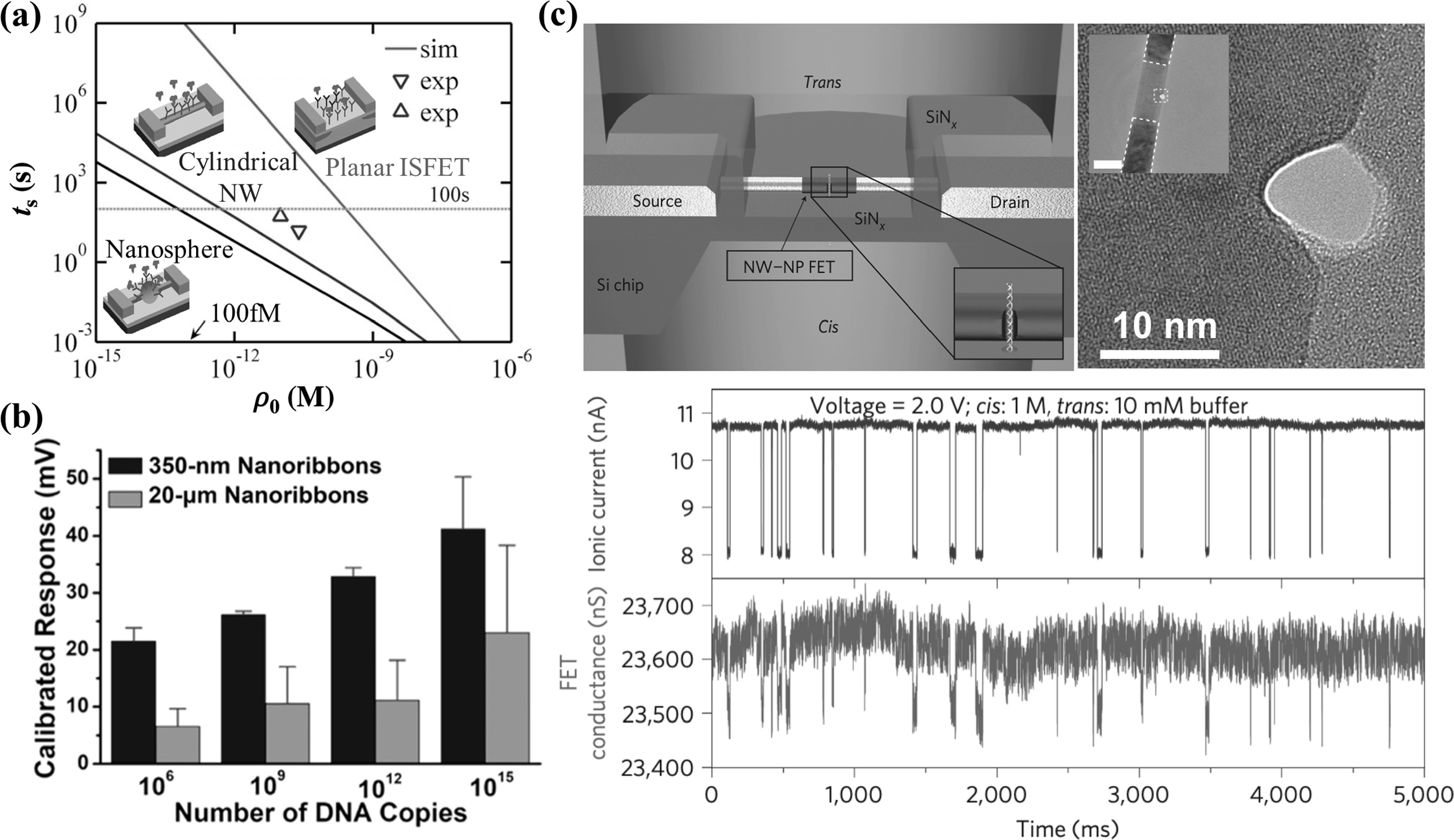
Advantages of nanostructured channels in field-effect biosensing. (a) Geometry of diffusion. Trade-off between the settling time *t*_s_ and detectable concentration *ρ*_0_ for planar ion-sensitive field-effect transistor (ISFET), cylindrical nanowire (NW), and nanosphere. For a settling time of 100 s, the cylindrical system can detect picomolar concentrations while the planar system can detect only in the nanomolar range. (b) Surface-to-volume ratio. Calibrated responses for complementary DNA hybridization for 350 nm versus 20 *μ*mwide In_2_O_3_ ribbon bioFETs. (c) Single-molecule characterization. Top left: schematic of the nanowire–nanopore measurement set-up. Inset: zoom-in view around the nanopore. NW–NP, nanowire–nanopore. Top right: high-resolution TEM image of a silicon nanowire with the nanopore off-axis at the nanowire edge. Inset: larger-scale TEM image of a nanowire–nanopore FET device showing the central silicon nanowire connected to darker NiSi contacts, which are indicated by the white dashed line. Scale bar (inset), 50 nm. Bottom: simultaneously recorded ionic current and FET conductance signals with 6 nM pUC 19 dsDNA in the *cis* chamber. Figures reproduced with permission from: (a) Reprinted from [72], with the permission of AIP Publishing; (b) Reprinted with permission from [75]. Copyright (2021) American Chemical Society; (c) Reproduced from [76], Copyright © 2011, Springer Nature Limited.

NanobioFETs have become popular since the first report on using silicon nanowires for detecting biological species [37]. Nanostructured channels offer three advantages over planar silicon channel in field-effect biosensing. First, nanostructured channels have a better geometry of diffusion. Cylindrical nanowires and nanospheres facilitate faster diffusion, resulting in enhanceddetection sensitivity compared to planar surfaces (figure 4(a)). For a settling time of 100 s, the cylindrical system can detect picomolar concentrations, while the planar system can only detect in the nanomolar range [72]. Second, nanostructured channels have a higher surface-to-volume ratio, leading to (i) improved electrostatic control of the channel conductance, (ii) a higher density of surface-bound analyte binding sites, and (iii) enhanced accessibility and analyte binding with convex surfaces [73] and reduced Debye screening with concave surfaces [74]. Figure 4(b) shows that 350 nm wide In_2_O_3_ ribbons are much more sensitive than 20 *μ*m wide ribbons [75]. Third, nanostructured channels enable single-molecule detection [76–80]. Analytes of interest, such as nucleic acids and proteins, are typically 1–10 nm in size. To characterize these individual biomolecules, biosensors need to have comparable feature sizes. Figure 4(c) shows the electrical detection of individual DNA molecules using a silicon nanowire FET-nanopore sensor [76]. As DNA molecules pass through a ∼10 nm nanopore one molecule at a time under a trans-membrane electric field, it blocks the nanopore channel, inducing a temporary drop in the ionic current. Meanwhile, the local electric field near the nanopore is altered, resulting in a decrease in the FET conductance.

**Figure 5.**
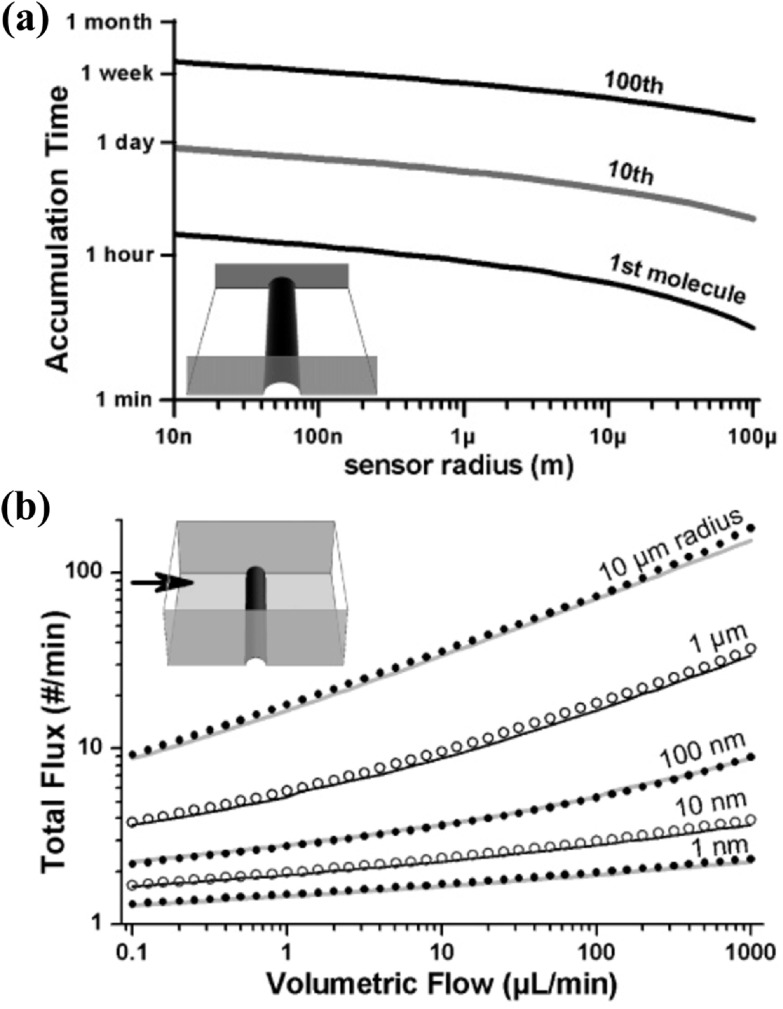
Limitations of nanometer-sized channels in field-effect biosensing. (a) Time required for a 10 *μ*m long hemi-cylindrical sensor to accumulate 1, 10, and 100 molecules by diffusion only. The inset shows the sensor geometry. The sensor lies at the bottom of a channel. The channel is 10 *μ*m wide and filled with a 1 fM analyte solution. (b) Total flux of molecules onto a hemi-cylindrical sensor in a microchannel under forced flow of analyte solutions. The sensor is 800 *μ*m long and with the stated radius, and the channel is 800 *μ*m wide and 100 *μ*m high. The analyte concentration is 1 fM. The points and the lines are the results of finite element analysis and analytical calculation, respectively. Figures reproduced with permission from: Reprinted with permission from [81]. Copyright (2005) American Chemical Society.

Unlike integrated circuits where smaller transistors indicate better electronic performance, smaller bioFETs do not necessarily result in higher detection sensitivity than larger ones. This is because the volume of sample fluids typically ranges from *μ*l to ml, corresponding to mm to cm length scales. Analyte molecules, such as nucleic acids and proteins, move slowly, with a diffusion length of 10–100 *μ*m within hours. For instance, when the analyte concentration is 1 fM, it takes over one hour for a 10 *μ*m long, 100 nm in radius hemi-cylindrical sensor to accumulate even the first analyte molecule by diffusion only (figure 5(a)). Flow-enhanced transport does not significantly improve the accumulation rate of analyte molecules by nanobiosensors (figure 5(b)). Consequently, within a detection time of minutes to hours, the sensing performance of nanobiosensors is limited by mass transport, not by signal transduction [81]. Large-area interfaces (*μ*m^2^–mm^2^) are needed to overcome mass transport limitations and achieve sub-fM LoD [81, 82]. Recent studies have shown that bioFETs with mm-sized channels are able to detect proteins with an LoD down to tens of zM [9, 10, 47]. In summary, a large-area but nanostructured channel maximizes the detection sensitivity of bioFETs.

## 4. Benchmarking the state-of-the-art bioFETs

**Figure 6.**
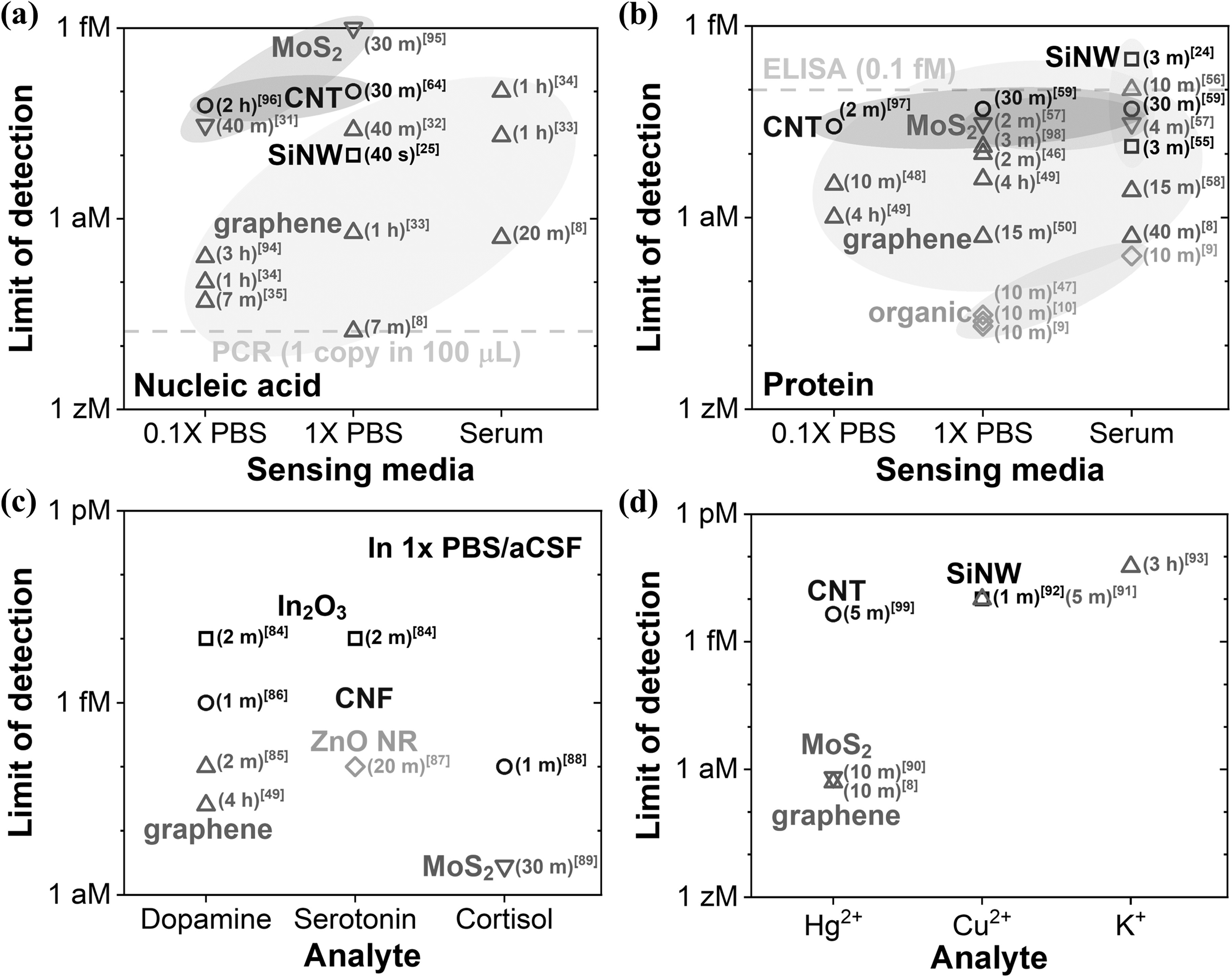
Benchmarking the limit of detection of bioFETs for different channel materials for the detection of (a) nucleic acids [8, 25, 31–35, 64, 94–96], (b) proteins [8–10, 24, 46–50, 55–59, 97, 98], (c) small biomolecules (dopamine [49, 84–86], serotonin [84, 87], and cortisol [88, 89]), and (d) ions (Hg^2+^ [8, 90, 99], Cu^2+^ [91, 92], and K^+^ [93]). For each data point, the detection time is shown in parentheses and the reference is shown in brackets. Only data points with sub-pM limit of detection were included. aCSF, artificial cerebrospinal fluid; CNF, cabon nanofiber; ZnO NR, ZnO nanorod. As a reference, PCR and ELISA technologies could detect nucleic acid and protein down to 1 copy per 100 *μ*l [11] and ∼0.1 fM [12, 13], respectively.

Benchmarking is essential for evaluating the performance of bioFETs. However, assessing all the figures of merit of a biosensor [83] is nearly impossible. In this review, we focus on the benchmark of the limit of detection (LoD), as shown in figure 6, which includes the detection time in parentheses.

Overall, the state-of-the-art bioFETs could detect nucleic acids down to 1 copy per 100 *μ*l in buffer [8] and 30–1000 copies per 100 *μ*l in serum [8, 33]. Similarly, they can detect proteins down to 1 copy per 100 *μ*l in buffer [9, 10, 47] and 15–30 copies per 100 *μ*l in serum [8, 9]. Small biomolecules like dopamine, serotonin, and cortisol can be detected with an LoD of 10–100 aM [49, 84–89], while Hg^2+^ can be detected with an LoD of ∼0.5 aM [8, 90], and Cu^2+^ and K^+^ with an LoD of ∼10 fM [91–93]. To our knowledge, no bioFET has achieved a sub-aM LoD for detecting small biomolecules at physiological ionic strength.

Innovations in bioFETs that have led to the sub-aM detection of analytes include: (1) the use of millimeter-sized crumpled graphene channels with 30 nm surface roughness [33, 49]; (2) millimeter-sized biofunctionalized gate [9, 10, 47]; (3) an increased chance of analyte recognition and binding using Y-shaped DNA dual probes [35] or multi-antibodies [50]; (4) the use of DNA nanostructures that bring captured analyte molecules within the Debye length upon negative liquid biasing for efficient signal transduction [8]; (5) the use of CRISPR-Cas13a coupled with graphene FETs that are stabilized with a hydrophobic coating [94]. We will discuss the physical mechanisms behind these innovations in the subsequent section.

It is noteworthy that 2D materials like graphene have achieved sub-aM LoD in field-effect biosensing for the detection of nucleic acids, proteins, and ions, whereas 1D materials such as SiNW and CNT have not yet reached sub-aM LoD, regardless of the analyte. Possible advantages of 2D materials over 1D materials in field-effect biosensing include atomically thin channel for superior electrostatic control by surface charges, and millimeter-sized channel for enhanced analyte capture.

We hypothesize that a useful detection time in point of care or point of use settings is less than 10 min, e.g. in a doctor’s office to wait for results. Even within this 10 min detection window, it is important to note that reported state-of-the-art bioFETs could detect nucleic acids down to 17 zM in artificial saliva [8], proteins down to 20 zM in 1× PBS and 250 zM in serum [9], dopamine [85] and cortisol [88] down to 0.1 fM in 1× PBS, serotonin down to 10 fM in 1× aCSF [84], Hg^2+^ down to 0.5 aM [8] and Cu^2+^ down to 10 fM [91, 92].

## 5. Improving sensitivity and limit of detection

According to IUPAC [100], sensitivity is the slope of the calibration curve, which represents the ratio of the change in signal to incremental change in analyte concentration or quantity. Limit of detection is recommended to be defined as the analyte concentration or quantity at which the signal equals three times the standard deviation of the signal from a suitable blank [101]. By this practice, limit of detection *c*_L_ is directly related to sensitivity *S* as three times the standard deviation of the blank measures *σ*_B_ over sensitivity, i.e. *c*_L_ = 3*σ*_B_/*S*. In this section, we will first discuss ways to improve the sensitivity of bioFETs, which in turn enhances the limit of detection. Then we will briefly discuss methods to reduce the background noise to improve the limit of detection. Next, we will discuss strategies to overcome the Debye limit at physiological ionic strength. Lastly, we will discuss the use of crumpled graphene bioFETs for detecting nucleic acids, proteins, and dopamine at physiological ionic strength and detecting DNA and SARS-COV-2 virus amplification.

### 5.1. Improving sensitivity by enhancing electrostatic control

**Figure 7.**
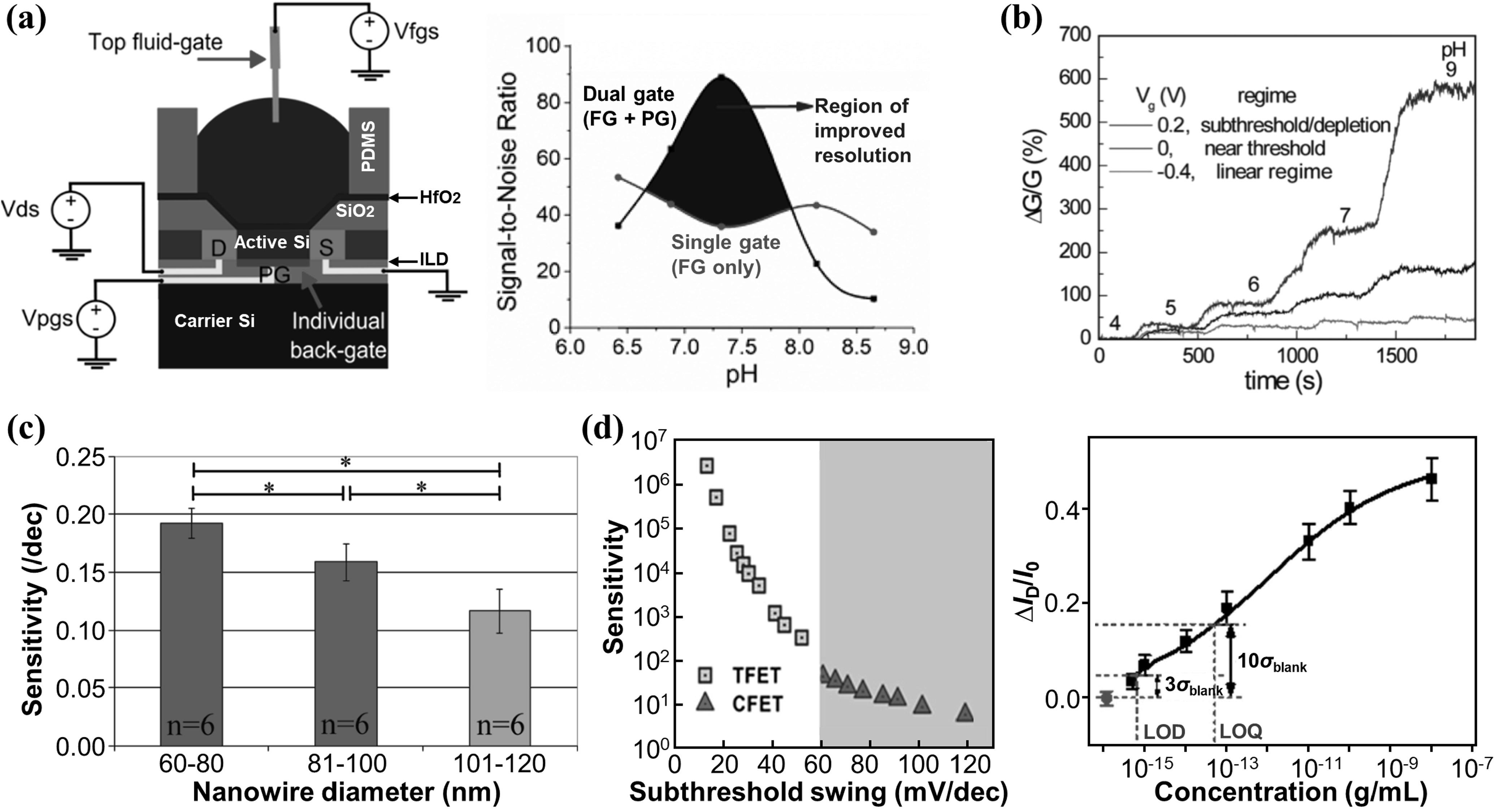
Improving sensitivity with enhanced electrostatic control. (a) Dual gating. Schematic showing the sensing setup of a dual-gated silicon FET biosensor, with the electrical connections for the source-drain (S–D, *V*_ds_), fluid gate (FG, *V*_fgs_), and poly gate (PG, *V*_pgs_) (Left). ILD denotes silicon dioxide interposing dielectric layer. Signal-to-noise ratio versus pH under tailored dual-gate (FG + PG) operation and standard single gate (FG only) operation (Right). (b) Subthreshold sensing: Relative changes in conductance, Δ*G*/*G*, for a p-type silicon nanowire FET in real-time pH sensing. (c) Reducing the diameter of nanowires. Device sensitivity of silicon nanowire FET biosensors for the detection of human immunoglobulin G proteins with different nanowire diameters (*n* = 6 for each group; *p < 0.05). (d). Tunneling based sensing. Sensitivity as a function of subthreshold swing for both conventional FET (CFET) and tunneling FET (TFET) based biosensors (Left). Relative changes in current, Δ*I*_D_/*I*_0_, as a function of the logarithm of CYFRA21-1 concentration for a silicon nanowire tunneling FET (Right). The limit of detection (LOD) and limit of quantitation (LOQ) equal to three and ten times of the standard deviation of blank response (*σ*_blank_). Figures reproduced with permission from: (a) Reprinted with permission from [105]. Copyright (2014) American Chemical Society; (b) Reprinted with permission from [107]. Copyright (2010) American Chemical Society; (c) Reprinted with permission from [113]. Copyright (2011) American Chemical Society; (d) (Left) Reprinted from [110], with the permission of AIP Publishing; (d) (Right) Reproduced from [55]. CC BY 4.0

Since bioFETs rely on the response of FET conductance to changes in surface potentials for signal transduction, it is intuitive to improve the sensitivity of bioFETs by enhancing the electrostatic control, as shown in figure 7. First, dual-gating has a broader window than a single gate for electrostatic control of channel conductance, which could be used to optimize the device sensitivity [102, 103]. For instance, the apparent sensitivity of a dual-gated silicon nanowire FET to pH can go beyond the Nernst limit of 60 mV pH^−1^ at room temperature [104]. The enhanced sensitivity increased the sensor’s signal-to-noise ratio, allowing the device to resolve smaller pH changes (figure 7(a)) [105]. A recent study used dual gating in ionic liquid to generate strong enough electric field with a strength up to 4.0 V nm^−1^ to modulate the bandgap of 2D materials [106], which could potentially be exploited to create ultrasensitive 2D bioFETs.

Second, operation in the subthreshold regime has the optimal sensitivity for bioFETs. Figure 7(b) shows the relative changes in conductance, Δ*G*/*G*, for a p-type silicon nanowire FET in real-time pH sensing. The FET operated in the subthreshold regime (gate bias *V*_g_ = 0.2 V) has better sensitivity than in the linear regime (*V*_g_ = −0.4 V) and near the threshold regime (*V*_g_ = 0 V) [107]. The subthreshold regime optimizes the gating effect of surface charges because, in this regime, the carrier density of silicon is low enough for the Debye screening length to be larger than the radius of the nanowire. Consequently, the entire volume of the nanowire is influenced by surface charges. Other studies on SiNW [65], CNT [108], and MoS_2_ [109] bioFETs also found optimal sensitivity in the subthreshold region.

Third, reducing the thickness of channel body, such as shifting from a planar silicon channel to atomically thin 2D material channel, enhances the electrostatic control. 2D materials are essentially surfaces and thus could be more sensitive than bulk 3D semiconductors to surface potential changes due to analyte binding. Another example is reducing the diameter of nanowires. Figure 7(c) shows the device sensitivity of silicon nanowire bioFETs for the detection of human immunoglobulin G proteins with different nanowire diameters. Narrower SiNWs achieve higher device sensitivity because a larger portion of the nanowire body is gated by surface charges when the nanowire is narrower.

Fourth, tunneling FET based biosensors could enable sub-thermionic sensing. Figure 7(d) (left) shows the potential of tunneling FET to improve sensitivity by up to four orders of magnitude over conventional FET [110]. Smaller subthreshold swing indicates a more significant change in drain current in response to variations in gate voltage, resulting in higher sensitivity to changes in surface charges. Conventional FET biosensors, limited by the thermionic carrier injection mechanism, are unable to achieve a subthreshold swing below 60 mV dec^−1^ at room temperature. In contrast, tunneling FET biosensors utilize band-to-band tunneling as a different current injection mechanism, allowing them to achieve a subthreshold swing below 60 mV dec^−1^. Consequently, tunneling FET biosensors overcome the sensitivity limitations of conventional FET biosensors. Figure 7(d) (right) shows the experimental demonstration of a silicon nanowire tunneling FET for the detection of CYFRA21-1 protein [55]. In this work, the subthreshold swing was 76 mV dec^−1^ and the LoD was 13 aM. Sub-aM LoD is possible by further reducing the subthreshold swing of the tunneling FET. In addition, subthreshold swing in heterojunction tunneling FETs is predicted to follow the trend of 3D–3D > 3D–2D > 2D–2D, because 2D confinement along the tunneling direction conserves momentum and energy and increases the tunneling probability [111]. Thus, 2D heterojunction-based tunneling FET biosensors are promising to deliver the smallest subthreshold swing [112] and, therefore, the highest device sensitivity.

### 5.2. Improving sensitivity by enhancing mass transport

**Figure 8.**
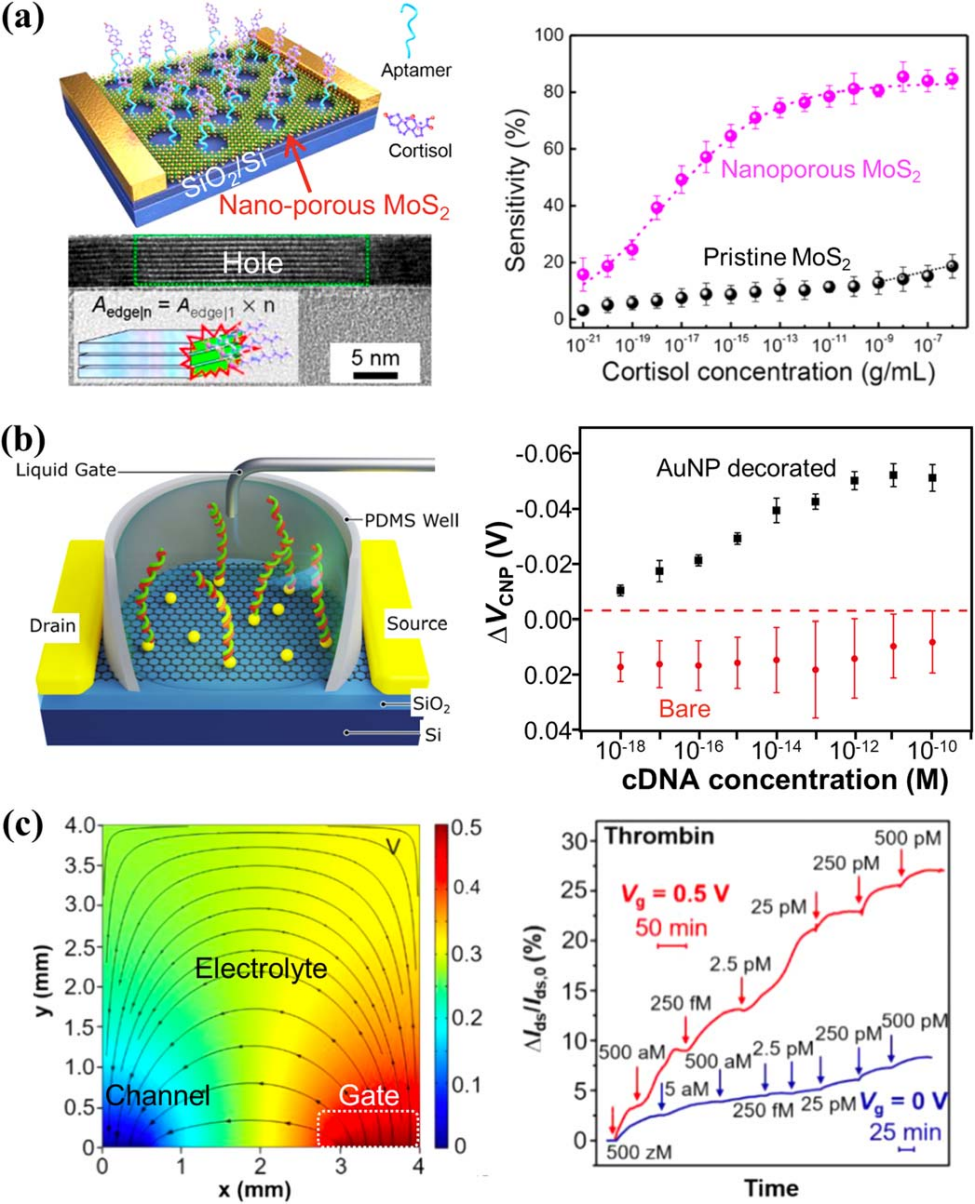
Improving sensitivity with enhanced mass transport. (a) Nano-structuring of micro-channels. Schematic of aptamer-functionalized nano-porous multilayer MoS_2_ FET biosensor for the detection of cortisol (Top Left). Cross-sectional STEM image showing the nanohole edge of multilayer MoS_2_ (Bottom Left). Device sensitivity as a function of the cortisol concentration for a pristine and a nano-porous MoS_2_ FET biosensor (Right). (b) Decorating micro-channels with conducting nanostructures. Schematic of a graphene FET biosensor decorated with Au nanoparticles (AuNP) for the detection of DNA (Left). The shift of charge-neutral point Δ*V*_CNP_ for AuNP decorated (black squares) and bare (red circles) graphene FET biosensors as a function of the concentration of complementary DNA (cDNA) to the aptamer (Right). (c) Electrostatic pre-concentration. Simulation of the electric field distribution at a gate bias *V*_g_ = 0.5 V (Left). Relative changes in drain-source current Δ*I*_ds_/*I*_ds,0_ of a graphene FET biosensor as a function of time upon addition of thrombin of increasing concentrations at *V*_g_ = 0 V and *V*_g_ = 0.5 V (Right). Figures reproduced with permission from: (a) Reprinted with permission from [89]. Copyright (2022) American Chemical Society; (b) Reprinted from [115], © 2020 The Authors. Published by Elsevier B.V; (c) Reprinted with permission from [116]. Copyright (2021) American Chemical Society.

Mass transport also affects the sensitivity of biosensors because it determines the amount of analyte detected by the biosensor within the detection time. Mass transport of analytes is typically limited by diffusion, which can be enhanced with increased fractal dimension of the channel [114]. For instance, planar microchannels can be nanostructured [89] or decorated with conducting nanoparticles or nanostructures [115], to increase their fractal dimension. Figure 8(a) shows aptamer-functionalized nano-porous multilayer MoS_2_ FET biosensors for the detection of cortisol. Planar multilayer MoS_2_ was shaped into a nanoporous structure using block copolymer lithography. The dangling groups on the nanoring edges of the nanopores were used for functionalization. Analyte molecules preferentially adsorbed onto the edge sites of the nanopores rather than on the basal plane exposed between them. This approach achieved a LoD of 1 ag mL^−1^ for cortisol with a detection time of 30 min. Figure 8(b) shows a graphene FET biosensor decorated with Au nanoparticles (AuNP) for the detection of DNA. The AuNP-decorated graphene sensor demonstrated high sensitivity to target DNA, detecting concentrations as low as 1 aM, while bare graphene remained insensitive to such low DNA concentrations. In addition, electrostatic pre-concentration is an effective way to overcome the diffusion limit and enhance the mass transport of analyte molecules [116]. Figure 8(c) (left) shows the electric field distribution at a gate bias *V*_g_ = 0.5 V. The electric field near the gate extends over 1 mm, which enriches the suspended charged analytes at the gate electrode. Figure 8(c) (right) shows the field-effect response of a graphene FET biosensor to thrombin of increasing concentrations at *V*_g_ = 0 V and *V*_g_ = 0.5 V. Electrostatic pre-concentration at *V*_g_ = 0.5 V results in several times higher sensitivity and over three orders of magnitude improvement in LoD.

Other ways to enhance mass transport in electronic biosensors include droplet evaporation to reduce the diffusion distance using nanotextured superhydrophobic electrodes [117], replacing analyte macromolecules by small molecules or ions via chemical reactions to increase the diffusion coefficient [14, 118], and dispersing magnetic nanoparticles throughout the sample solution to capture the analyte and then collecting the nanoparticles with a magnet for detection [119, 120]. For instance, graphene bioFETs have been combined with enzymatic reaction to detect urease and the gastric cancer pathogen Helicobacter pylori at physiological ionic strength [118]. It overcame the limitation of Debye screening by detecting ammonia as an enzymatic reaction product from urease. Two hundred seventy zeptomoles of biotinylated urease and Helicobacter pylori corresponding to 0.04 bacterial cells were successfully detected within 30 min. Convection is not an effective means of enhancing mass transport for surface-based biosensors [121], such as bioFETs, and is thus not recommended.

### 5.3. Improving sensitivity by enhancing biorecognition and binding

**Figure 9.**
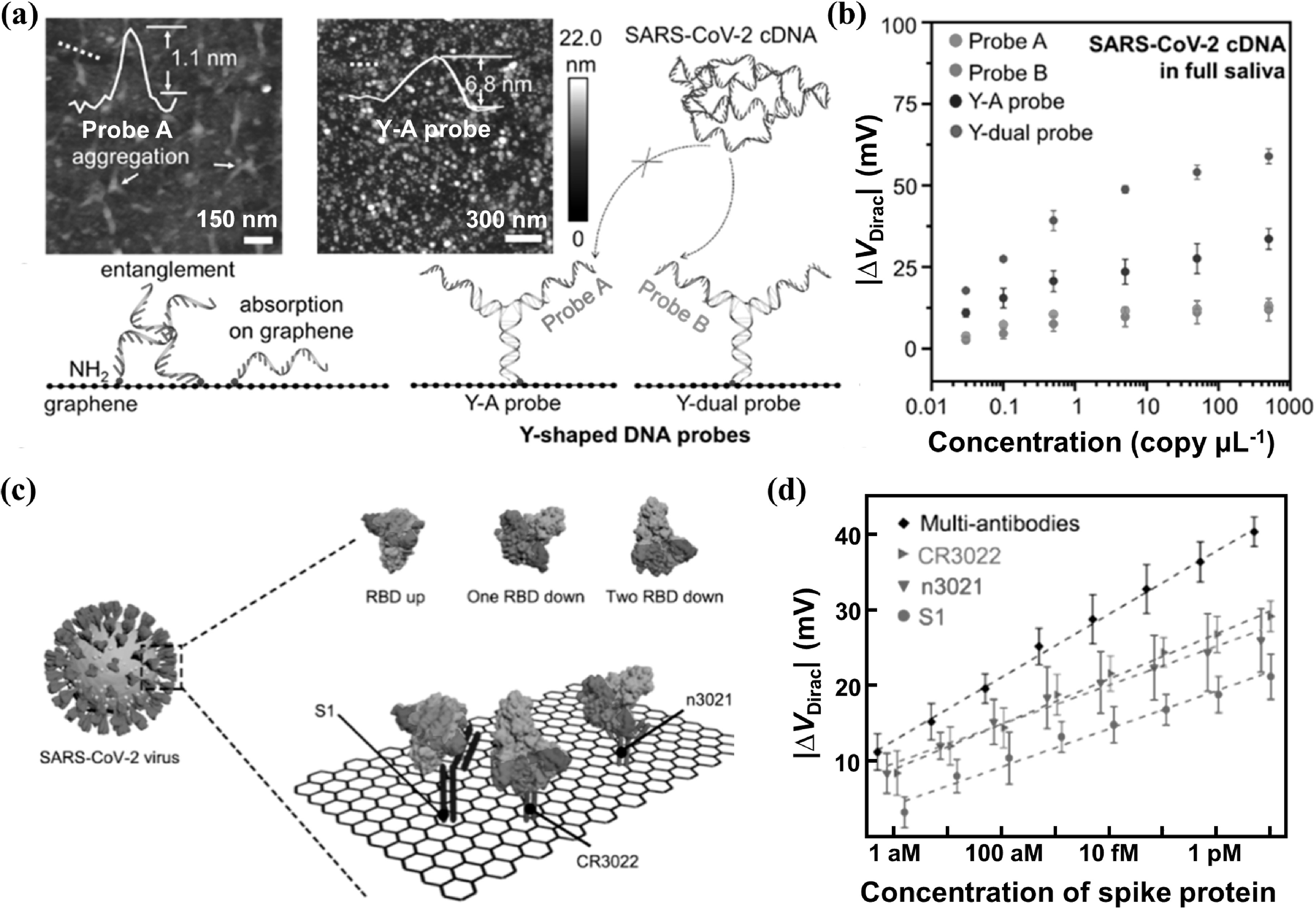
Improving sensitivity with enhanced biorecognition and binding. (a) AFM images of graphene modified with ss-DNA probes (probe A) or Y-shaped DNA probes (Y–A probe) and schematics of the sensing interface of a graphene FET biosensor modified with ss-DNA probes or Y-shaped DNA probes. (b) Dirac point shift Δ*V*_Dirac_ of a graphene FET biosensor with different probes as a function of SARS-CoV-2 cDNA concentration from 0.03 to 500 copy *μ*l^−1^ in 100 *μ*l of full artificial saliva. (c) Schematic of SARS-CoV-2 virus binding events on the graphene surface. The spike protein is present in three spatial orientations, including the ‘RBD up’, ‘one RBD down’, and ‘two RBD down’ configurations. (d) Comparison of the device sensitivity using multi-antibodies and single antibody CR3022 (red), n3021 (green), and S1 (blue). Figures reproduced with permission from: (a)–(b) Reprinted with permission from [35]. Copyright (2021) American Chemical Society; (c)–(d) Reprinted with permission from [50]. Copyright (2021) American Chemical Society.

In addition to signal transduction and mass transport, biorecognition and binding also affect the sensitivity of biosensors. A higher biorecognition ratio and stronger binding can result in more binding events and a larger signal. For instance, peptide nucleic acid (PNA) probes exhibit a stronger binding affinity to analyte nucleic acid than DNA probes, resulting in higher sensitivity [34]. This is due to the absence of electrostatic repulsion between the charge-neutral backbone of PNA and the negatively charged backbone of analyte nucleic acid. In figure 9, we present two recent examples of how biorecognition and binding can be enhanced to improve sensitivity.

Figures 9(a) and (b) show the use of graphene bioFETs functionalized with Y-shaped DNA dual probes for detecting SARS-COV-2 nucleic acid [35]. Y-shaped DNA probes offer a higher biorecognition ratio than ssDNA probes because the latter tend to aggregate and entangle due to their structural flexibility and lie flat on graphene surface due to π−π stacking interactions between nucleosides and graphene [122]. In contrast, the rigid stem structure of Y-shaped DNA probes keeps them upright at the surface without aggregation, as confirmed by atomic force microscopy (AFM) in fluid in figure 9(a). Other rigid DNA nanostructures, such as tetrahedral DNA, also show improved biorecognition compared to ssDNA probes [8, 116, 123]. Moreover, Y-dual probes with two different recognition sites have a higher sensitivity than Y–A probes with two identical recognition sites (figure 9(b)), suggesting dual probes facilitate biorecognition and binding.

Figures 9(c) and (d) show the use of graphene bioFETs modified with multi-antibodies for the detection of SARS-COV-2 spike S1 proteins with sub-aM LoD [50]. Multi-antibodies (CR3022, n3021 and S1) can bind not only to the receptor binding domain (RBD), but also to adjacent sites of the spike protein, as illustrated in figure 9(c). This cooperative recognition enables binding with different spatial configurations and increases the binding affinity, thus improving the sensitivity.

### 5.4. Improving limit of detection by minimizing background noise

To achieve a low limit of detection, especially in physiological fluids, a biosensor with high sensitivity should also minimize the background noise, as follows. First, minimize non-specific binding of parasitic molecules to receptors that are immobilized on the sensor surfaces. Sequence-specific hybridization can discriminate single nucleotide polymorphisms in nucleic acid detection with an LoD of 25 aM [32]. Second, block un-passivated regions of the channel surface to minimize adsorption of parasitic molecules. Ethanolamine, bovine serum albumin (BSA), and 6-mercapto-1-hexanol (MCH) are commonly used as blocking agents after immobilization of receptors [124]. Third, block non-sensing region of the sensor surface. If target molecules are captured by the receptors on the surrounding substrate rather than at the channel surface, the sensitivity will be dramatically reduced [125]. The contact region also needs to be passivated for electrostatic gating dominated sensing, which is a more reliable sensing mechanism [126]. Fourth, reduce sensor drift [127]. One notable example is that hydrophobic graphene often has poor adhesion to hydrophilic oxide surfaces and results in significant sensor drift. Coating the substrate with a hydrophobic layer can effectively mitigate the drift [94].

### 5.5. Overcoming Debye limit at physiological ionic strength

Debye screening is a fundamental limit to field-effect biosensing [128, 129]. The Debye length at physiological ionic strength (∼0.1 M) is just 1 nm, whereas aptamers are about 25–80 bases long [130] (i.e. 3–5 nm in radius of gyration [131]), and antibodies are about 10 nm in size [132]. While one can dilute clinical samples to lower the ionic strength and hence increase the Debye length, it also lowers the analyte concentration, making it more difficult to detect analytes of low abundance. Another approach is to extract and purify analytes from clinical samples and measure in a dilute buffer. However, significant sample preparation is needed, and some loss of analytes cannot be avoided. Moreover, molecular affinity and specificity are reduced without stabilizing salts [3]. Finally, it is important to monitor biological processes in physiological fluids because these processes are highly dependent on the presence and concentration of ions in the environment [133].

**Figure 10.**
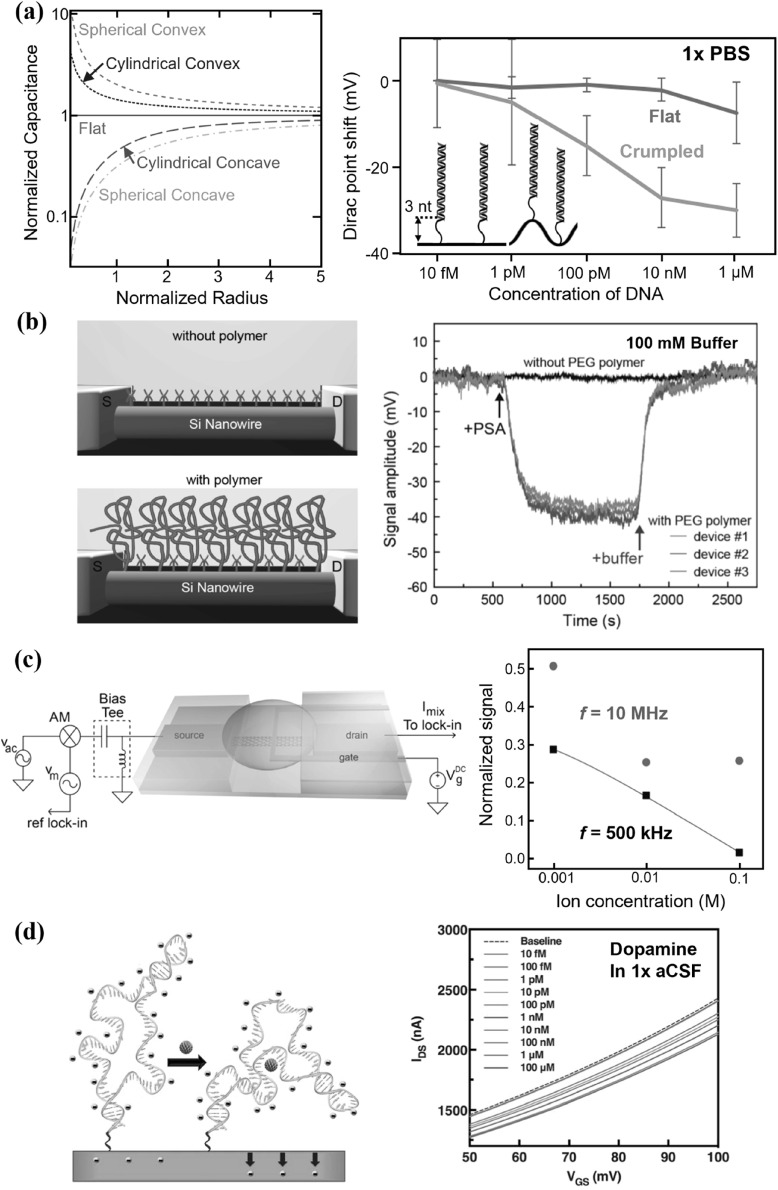
Overcoming Debye limit at physiological ionic strength. (a) Plot of electrolyte capacitance density versus radius of curvature of electrode (Left). The radius is normalized to the Debye length, and the capacitances are normalized to that of the flat electrode. Dirac point shift of a crumpled graphene FET biosensor versus flat graphene for the detection of a 19 nt target DNA by DNA hybridization with 22 nt probe DNA (Right). Hybridized dsDNA was 3 nt away from the surface. (b) Schematic of a SiNW FET biosensor without (top) and with (bottom) a porous and biomolecule permeable polymer polyethylene glycol (PEG, green) modification (left). Nanowires are modified with APTES receptors (magenta) to capture prostate-specific antigen (PSA). Signal amplitude versus time for APTES modified SiNW FETs following the addition of 100 nM PSA and pure buffer (Right). (c) Schematic of a mixing current measurement setup of a carbon nanotube FET biosensor with an amplitude-modulated (AM) high-frequency input signal at the source electrode (Left). Sensor response with varying background ionic strengths at *f* = 500 kHz and *f* = 10 MHz (Right). (d) Stem-loop aptamers reorient closer to FETs within or near the Debye length to deplete channels electrostatically (Left). Exposure of dopamine aptamer–FETs to dopamine (1× aCSF) led to concentration-dependent reductions in source-drain currents (Right). Figures reproduced with permission from: (a) (Left) Reproduced with permission from [74]; (a) (Right) Reproduced from [33]. CC BY 4.0; (b) Reprinted with permission from [134]. Copyright (2015) American Chemical Society, © 2015 ACS; (c) Reprinted with permission from [136]. Copyright (2012) American Chemical Society, © 2012 ACS; (d) From [84]. Reprinted with permission from AAAS.

Over the past decade or so, four general approaches have emerged to overcome Debye limit at physiological ionic strength. First, electrolytes near concave surfaces have increased Debye length compared to near flat surfaces. Figure 10(a) (left) illustrates that concave surfaces have smaller electrolyte capacitances than flat surfaces [74], suggesting a larger Debye length near concave surfaces. Figure 10(a) (right) shows that the detection of DNA hybridization 3 nt (1 nm) away from the flat graphene surface is not possible since the Debye length near the flat surface is 1 nm in 1× PBS [33]. However, DNA hybridization can be detected with crumpled graphene, because the Debye length near the concave surfaces of the graphene crumples is larger than 1 nm.

Second, coating the channel surface with a dense, partially hydrated nano-porous film, such as polyethylene glycol (PEG) [134] or polyelectrolyte multilayers (PEM) can overcome Debye limit at physiological ionic strength [135]. The entropic cost of confining ions inside the film increases the screening length [135]. Figure 10(b) illustrates the use of PEG coated SiNW bioFET for the detection of 100 nM prostate-specific antigen (PSA) in 100 mM buffer, which is not possible without PEG coating [134].

A third approach is to disrupt the electric double layer through high-frequency perturbation. At direct current or low frequencies (<1 MHz), ions in solution migrate under electric field and form the EDL; at high-enough frequencies (⪆10 MHz), the alternative current driving force can no longer overcome the solution drag and hence ions do not have sufficient time to form the EDL to screen [136]. Figure 10(c) shows the detection of streptavidin binding to biotin in 100 mM buffer at a frequency of 10 MHz using a CNT bioFET [136]. However, by disrupting the EDL, high-frequency signals can penetrate deeper into the solution [137], which potentially increases the background noise and limits the LoD and selectivity of bioFETs.

Fourth approach is to live with Debye screening and sense within the electric double layer. For instance, while the size of aptamers exceeds 1 nm, they can be designed to reorient toward the sensor surface within or near the Debye length upon analyte binding, leading to electrostatic depletion of the channel (figure 10(d)) [84]. Another example uses size-reduced antibody fragments as receptors [138]. While the whole IgG antibody measures 9–10 nm in size, the Fab fragment, comprising only the antigen-binding part, has a reduced size of 2–3 nm. By carefully engineering the linker’s flexibility and density, size-reduced antibody fragments enable biorecognition events to occur in closer proximity to the nanowire surface, falling within the Debye screening length. Furthermore, if the analyte generates reaction products that freely diffuse and reach the sensor surface, then the detection is independent of Debye screening, as seen in the case of the urease-ammonia gas reaction used for bacteria detection [118].

### 5.6. Crumpled graphene FET biosensors

**Figure 11.**
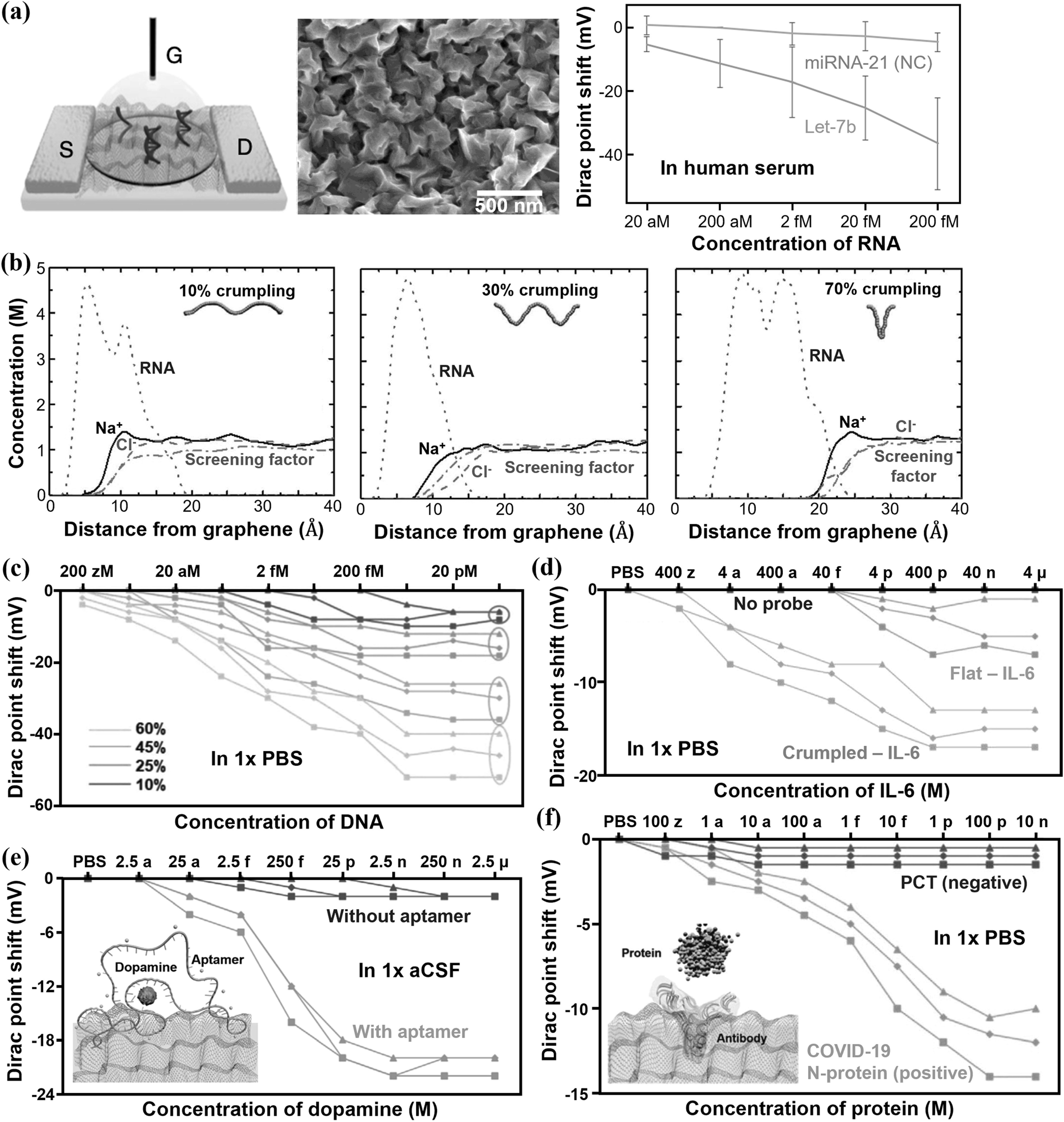
Crumpled graphene FETs for the detection of nucleic acids, dopamine, and proteins at physiological ionic strength. (a) Schematic of a crumpled graphene FET biosensor (Left), SEM image of crumpled graphene (Middle), and Dirac point shift of a crumpled graphene FET biosensor for the detection of the target miRNA let-7b in human serum by DNA hybridization (Right). (b) Molar concentration of ions (sodium and chloride) and the backbone of COVID-19 RNA strand segment along with the screening factor of ions as a function of the distance from the graphene surface with different crumpling ratios. (c) Dirac point shift by ssDNA adsorption on graphene FET sensors with various crumpling ratios in 1× PBS. Dirac point shift of the sensor with the detection of (d) IL-6 protein in 1× PBS, (e) dopamine in artificial cerebrospinal fluid (aCSF), and (f) COVID-19 N-protein in 1× PBS. Figures reproduced with permission from: (a) Reproduced from [33]. CC BY 4.0; (b)–(f) [49] John Wiley & Sons. © 2021 Wiley-VCH GmbH.

Among the techniques to surpass the limit of Debye screening, creating rough sensor surfaces and hence concave surfaces is a universal solution that can be applied to detect various analytes. Rough surfaces also facilitate analyte transport by improving diffusion with increased fractal dimension [114] and by enhancing evaporation-induced convection [139]. Figure 11 shows the use of crumpled graphene bioFETs for the detection of nucleic acids, proteins, and dopamine under physiological ionic strength. The crumpled graphene was obtained by annealing flat graphene on a pre-strained polystyrene substrate, which caused buckling of graphene due to the shrinkage of the substrate [140]. Figure 11(a) shows the detection of target RNA let-7b in human serum sample, down to 20 aM, corresponding to ∼600 nucleic acid molecules [33]. Simulation results in figure 11(b) show that the effective Debye length increases with the crumpling ratio [49]. Figure 11(c) shows the Dirac point shifts by ssDNA absorption on graphene FET sensors with various crumpling ratios. As expected, more crumpled graphene FET sensors produce larger Dirac point shifts. Figures 11(d) and (f) show the detection of IL-6 protein and COVID-19 N-protein in 1×PBS, down to 4 aM and 10 aM, respectively. Finally, figure 11(e) shows the detection of dopamine in artificial cerebrospinal fluid, down to 25 aM.

**Figure 12.**
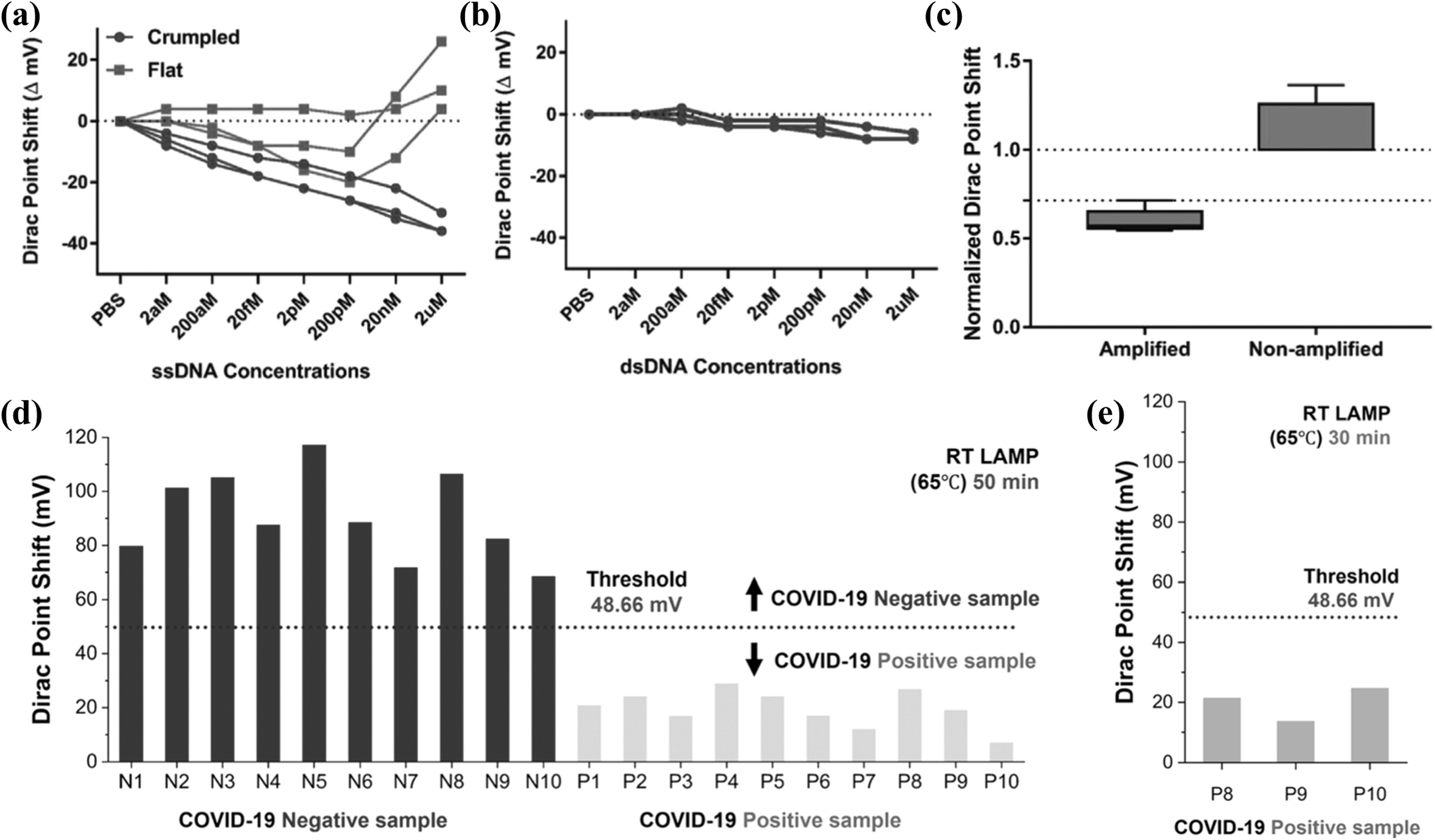
Crumpled graphene FET based detection of DNA and SARS-COV-2 virus amplification. (a) Dirac point shift of the FET sensor for ssDNA adsorption on flat and crumpled graphene. (b) Dirac point shift of the crumpled graphene FET for dsDNA adsorption. (c) Box and whisker plot for the normalized Dirac point shift for amplified and non-amplified DNA samples. (d) Bar plot of the Dirac point shift of 10 known positive and 10 known negative COVID-19 clinical samples (RT-LAMP; 65 °C, 50 min) on crumpled graphene FETs. (e) Bar plot of the Dirac point shift of 3 known positive clinical samples with the lowest viral load (RT-LAMP; 65 °C, 30 min) on crumpled graphene FETs. Figures reproduced with permission from: (a)–(c) [142] John Wiley & Sons. © 2020 WILEY-VCH Verlag GmbH & Co. KGaA, Weinheim; (d)–(e) Reprinted with permission from [143]. Copyright (2021) American Chemical Society.

Crumpled graphene FETs can also be used to detect enzymatic amplification by monitoring the reduction in primer (ssDNA) concentration in a reaction. Figure 12(a) shows unidirectional and distinct Dirac point shift of crumpled graphene with increasing ssDNA concentrations from 2 aM to 2 *μ*M, while dsDNA induces negligible Dirac point shift, as shown in figure 12(b). This is because ssDNA binds strongly onto graphene due to the π−π stacking interactions between graphene and the aromatic ring structure of unpaired nucleobases, whereas dsDNA lacks unpaired nucleobases for such strong interactions [141]. Figure 12(c) shows that negative samples can be clearly distinguished from positive samples after amplification, with an LoD down to 4 aM [142]. Moreover, crumpled graphene FETs, combined with reverse transcriptase loop-mediated isothermal amplification (RT-LAMP), can detect the SARS-CoV-2 virus in clinical samples ranging from 10 to 10^4^ copies/*μ*l [143]. Based on the Dirac point shift, these devices can differentiate between positive and negative clinical samples in 30–50 min (figures 12(d) and (e)).

## 6. BioFET array

In addition to ultrahigh sensitivity, parallelized and multiplexed detection is yet another promise of bioFETs. First, the use of large bioFETs array can improve the sensitivity of the assay. By collecting large, statistically meaningful data sets from multiple sensors on each analyte, it is possible to increase the signal-to-noise ratio and improve measurement accuracy through cross validation of data [15, 16]. Second, massive parallelization also improves the reliability of the assay by providing redundancy. If one or more sensors in the array fail or give anomalous readings, the data from the other sensors can be used to compensate and ensure the accuracy of the overall assay result. Additionally, it is possible to designate a specific region of the array as a negative control to monitor real-time drift. This negative control can then be used to compensate the drift effects of the entire array [15, 17]. Third, the use of bioFETs array allows for simultaneous detection of multiple analytes in a single sample [20, 144–147], increasing the throughput of the assay. This is particularly important in clinical applications, where it is necessary to analyze large number of samples quickly and accurately.

**Figure 13.**
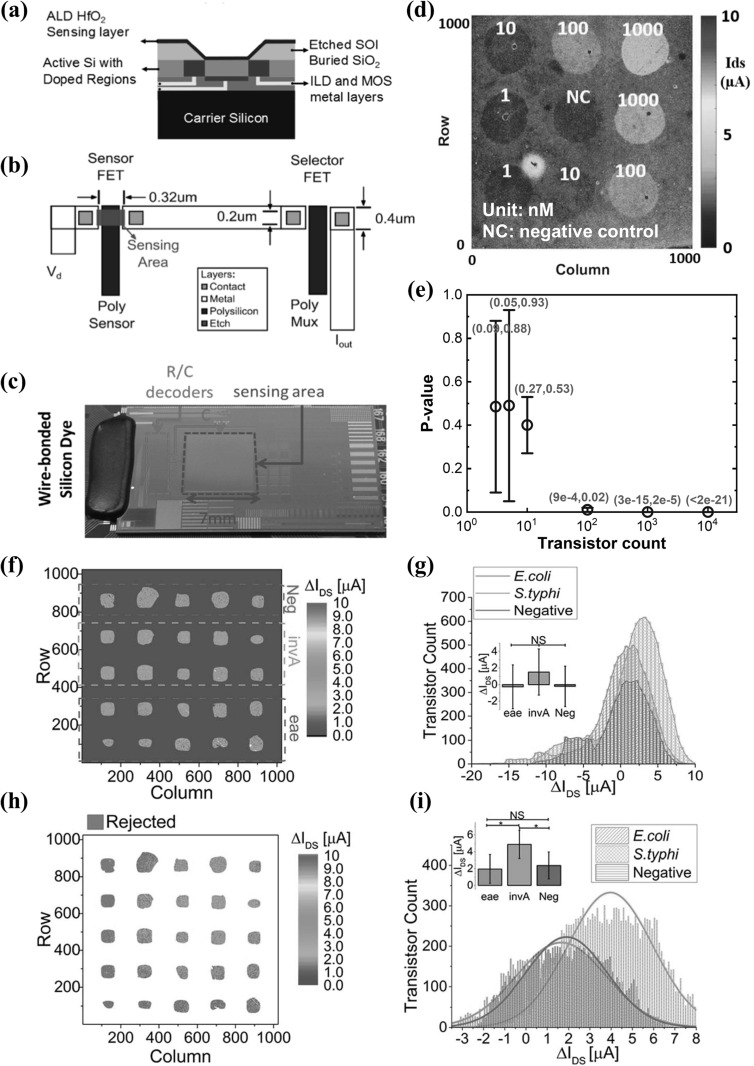
Dual-gated ISFET biosensor in one-million array. (a) Cross section of the sensing element. (b) Top view of pixel schematic in the array. (c) Photograph of the dual-gate ISFET array, showing the 7 × 7 mm^2^ sensing area, decoding portions, and wire-bonds to PCB. (d) Heat map of drain current with different concentrations of target miRNA-let7 in the array. (e) *p*-value graph comparing the drain current of 1 nM target miRNA-let7 against that of negative control as a function of transistor count. The error bars are the maximum and the minimum. The exact range of *p*-values is shown in brackets in red. (f) Differential drain current map for parallel detection of foodborne pathogens. Eae and invA are the target genes for the detection of *E. coli O157* and *S. typhi* respectively. Neg denotes negative control. (g) Unfiltered drain current distributions for the sensing bioFETs in each group of chambers. (h) Differential drain current map with discarded sensors in pink and non-sensing devices (outside the wells) in white. (i) Filtered drain current distributions for each group of chambers. The inset bar plots in (g) and (i) show mean, standard deviation, and statistical significance between data groups. Figures reproduced with permission from: (a)–(c) Reprinted from [16], © 2017 Elsevier B.V. All rights reserved, (d)–(e) Reproduced from [17], Copyright © 2018, Springer Science Business Media, LLC, part of Springer Nature; (f)–(i) Reproduced from [15] with permission from the Royal Society of Chemistry.

Figure 13 shows an example bioFET array platform with one million dual-gated ion-sensitive field-effect transistors (ISFETs), which was fabricated with a complementary metal-oxide-semiconductor (CMOS) process by TSMC [105]. As illustrated in figure 13(a), each transistor has an individually addressable back gate and a gate oxide that is directly exposed to the solution [16]. Utilizing on-chip integrated circuits for row and column addressing and a PXI IC tester to measure signals (figures 13(b) and (c)), the drain current of each dual-gated ISFET sensor in the array can be serially acquired in just 90 s [16]. Figure 13(d) shows the detection of different concentrations of target nucleic acid molecules in the array based on this one million biosensor array platform [17]. A PDMS well with 9 holes provided isolated reaction chambers, with each chamber containing ∼15 000 bioFETs. All the *p*-values from t-tests between each concentration are less than 0.0001, demonstrating that this array sensor is highly reliable and robust against noise artifacts. Figure 13(e) shows the effect of transistor count on the *p*-value based on randomly selected pixels. For experiments with low number of transistors, the *p*-value is high and variable. However, as the transistor count per reaction increases, particularly above a few hundred transistors, the *p*-value becomes very low for all tests, suggesting that the system becomes highly reliable and robust. Figures 13(f)–(i) demonstrate parallel detection of foodborne pathogens using the same bioFETs array platform [15]. The raw differential current distribution data in figure 13(g) shows non-statistically significant results between samples. By using redundancy techniques to minimize the overall standard deviation, the Grubbs test to eliminate measurements outside the expected normal distribution, and reference micro-chambers to subtract the common noise, new current distributions presented in figure 13(i) show statistically significant differences (*p* < 0.05) between invA and the other two groups.

**Figure 14.**
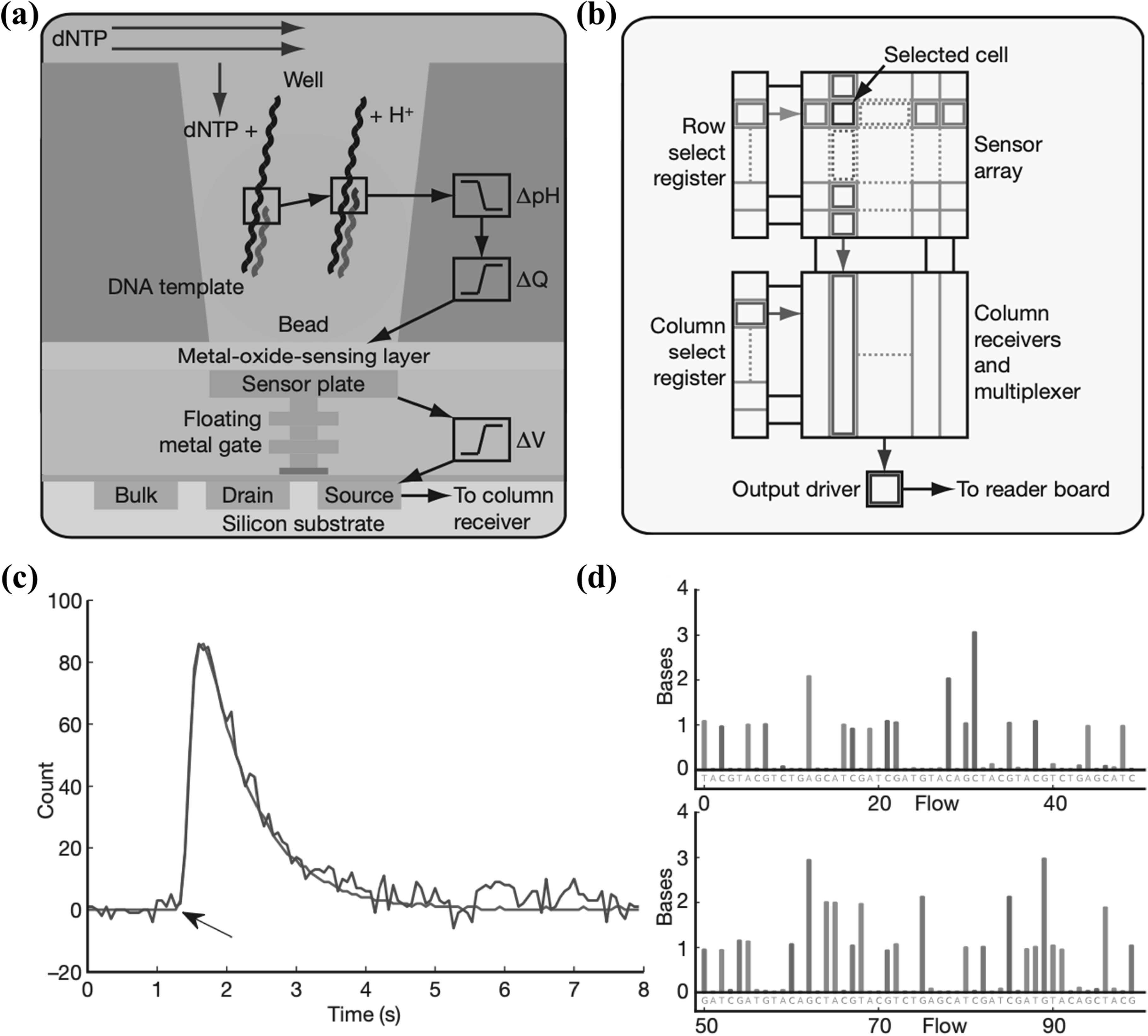
Ion Torrent sequencing. (a) Schematic of a well, a bead containing DNA template, and the underlying sensor and electronics. Protons (H^+^) are released when nucleotides (dNTP) are incorporated on the growing DNA strands, changing the pH of the well (ΔpH). This pH change induces a change in surface potential of the metal-oxide-sensing layer, and a change in potential (Δ*V*) of the source terminal of the underlying field-effect transistor. (b) Sensors are arranged in a two-dimensional array. A row select register enables one row of sensors at a time, causing each sensor to drive its source voltage onto a column. A column select register selects one of the columns for output to external electronics. (c) 1-nucleotide incorporation signal from an individual sensor well; the arrow indicates start of incorporation event, with the physical model (red line) and background corrected data (blue line) shown. (d) The first 100 flows from one well. Each coloured bar indicates the corresponding number of bases incorporated during that nucleotide flow. Reproduced with permission from [14]. Copyright © 2011, The Author(s)  CC BY-NC-SA 3.0.

Another notable example of bioFETs array is the CMOS integrated circuit-based DNA sequencing, used by Ion Torrent [14], as illustrated in figure 14. Each chip contains 1.2 million individual wells, allowing for parallel and simultaneous detection of independent sequencing reactions. As shown in figure 14(a), each well contains one bead functionalized with DNA templates and one ISFET pH sensor at the bottom. Sequencing primers and DNA polymerase are bound to the templates. During ion torrent sequencing, all four nucleotides are introduced sequentially into the wells in an automated run. When the incoming nucleotide complements the template base downstream of the sequencing primer, it is incorporated into the nascent strand by the bound polymerase, increasing the primer length by one base. The hydrolysis of the incoming nucleotide triphosphate releases a single proton, causing a proportional pH shift (0.02 pH units per base incorporation) detected by the FET pH sensor at the bottom of the well. This shift is digitized and converted to voltage by off-chip electronics. The signal generation and detection occur over 4 s, as shown in figure 14(c). A wash (∼0.1 s) is used after each flow to eliminate remaining nucleotides. A typical 2 h run using an Ion Torrent chip with 1.2 million sensors generates approximately 25 million bases.

## 7. Reusable bioFETs

While bioFETs can be made for single uses, reusable bioFETs would be much more cost-effective, making them more commercially viable for clinical or research applications. Furthermore, real-time reusable (or reversible) bioFETs could be incorporated into wearable or implantable systems to monitor analyte concentrations continuously *in vivo* [148–154], with potential applications for disease diagnosis, drug discovery, and personalized medicine.

**Figure 15.**
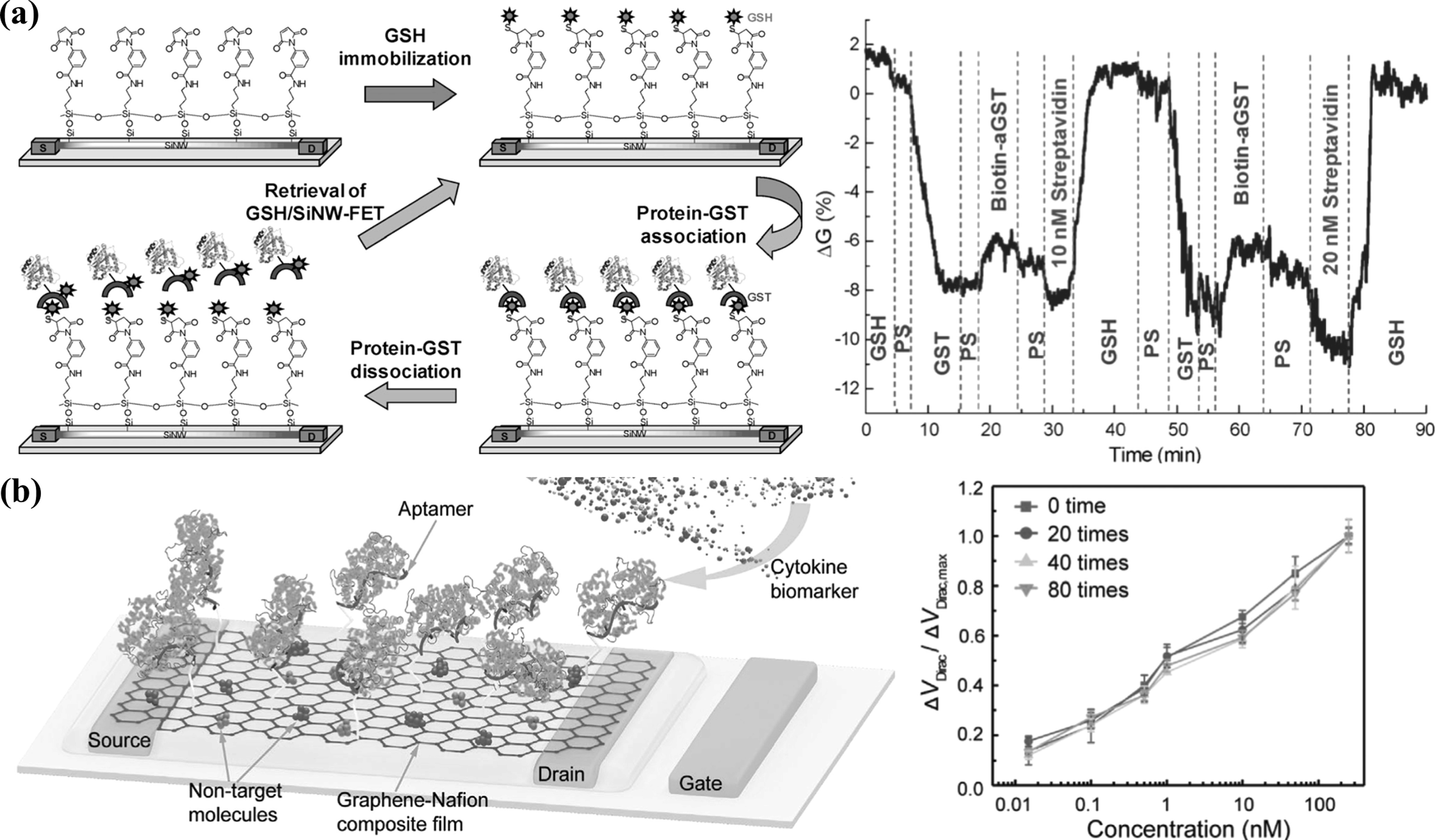
Examples of reusable bioFETs. (a) Schematic for the reversible binding of protein-GST association on a silicon nanowire bioFET (Left). The nanowire is first modified with APTMS and MBS linkers, and then immobilized with GSH. A particular protein-GST binds with GSH for protein detection. At the end of each measurement, captured protein-GSTs are removed with 10 mM GSH solution to retrieve the bioFET. Real-time detection of the binding of biotin-aGST to GST/GSH/SiNW bioFET and subsequent sensing of streptavidin (Right). (b) Schematic of the aptamer graphene-Nafion bioFET for cytokine detection (Left). Detection of IFN-γ protein using the biosensor with different regenerative cycles (Right). Ethanol was used to dissolve the Nafion film to regenerate the bioFET. Figures reproduced with permission from: (a) Reprinted from [158], Copyright © 2009 Elsevier Ltd. All rights reserved; (b) [171] John Wiley & Sons. © 2020 Wiley-VCH GmbH.

BioFETs can theoretically be regenerated by either dissociating captured analyte from receptors (as shown in figure 15(a)) or removing surface-immobilized receptors altogether (as shown in figure 15(b)). The latter approach is less favourable as it adds cost and effort to re-functionalize sensor surfaces with receptors every time. Table 1 summarizes reusable bioFETs that have been reported in the literature. With reversible analyte–receptor binding, bioFETs can operate continuously without regeneration [152, 155], or they can be restored with a simple buffer or DI water rinse [37, 156, 157] or a mild electrolyte (1–10 mM) bath [158, 159]. However, reversible binding limits sensitivity and makes sub-pM detection challenging [160]. For sensors with strong analyte-receptor binding, the solvent environment can be altered to weaken the interaction to allow for dissociation and regeneration, with reagents such as acid buffers (pH = 2–4) [161, 162] and detergents [163]. In particular, aptamers can be denatured with 6 M guanidinium chloride and refolded multiple times without loss of activity [164, 165]. Strong acid buffers (pH = 1–2) [166, 167] and solutions such as 8 M urea [168, 169] and 0.5 M DTT [170] can completely remove the biofunction layer.

**Table 1.** Reusable bioFETs.

**Channel**	**Analyte**	**Receptor**	**Regeneration technique**	**# cycles**	**Signal loss between cycles**	**LoD**	**Sensing media**	**References**
**Without removal of receptors**								

SiNW	protein-GST association	GSH	10 mM GSH	6	∼5%	0.5 nM	0.1× PS	[158]
SiNW	protein-GST association	GSH	1 mM GSH	2	—	7 nM	0.1× PS	[159]
SiNW	biotin; Ca^2+^	m-antibiotin; calmodulin	buffer	—	—	—	5 mM NaCl	[37]
SiNW	glucose	glucose oxidase	liquid gating	—	—	0.15 mM	1× PBS; blood	[150]
CNT	dopamine	carboxyphenyl boronic acid	10 mM HCl	5	4.8%	1 pM	10 mM PBS	[161]
P3HT	Na^+^	Na^+^ selective membrane	reversible	5	∼3%	1 *μ*M	salt solution	[155]
diamond	PDGF protein	aptamer	surfactant	4	—	—	1 mM NaCl	[163]
graphene	thrombin protein	aptamer	buffer	—	—	10 nM	5 mM MES	[156]
graphene	MMP-9 protein	IgG	reversible	—	—	8 pM	tear	[152]
graphene	glucose	AAPBA	0.1 mM HCl	20	0.63%	1.9 *μ*M	1× PBS; urine	[162]
graphene	PSA	aptamer	6 M guanidinium chloride	2	—	2 nM	1× PBS with 2 mM Mg^2+^	[165]
WSe_2_	glucose	glucose oxidase	DI water	2	35%	1 mM	DI water	[157]

**With removal of receptors**								

planar Si	dopamine	Fe_3_O_4_@AuNPs	ultrasonication	15	0.33%	3.3 nM	100 mM PBS	[172]
SiNW	streptavidin	biotin	*β*-CD solution	5	—	—	1 mM Na_2_CO_3_	[173]
SiNW	HbA1c protein	aptamer	DTT redox agent	4	<3%	0.2 nM	1× PBS	[170]
SiNW	streptavidin	PLL-biotin copolymer	pH = 2 buffer	2	—	—	—	[166]
CNT	IL-4, IL-10 proteins	antibody-functionalized magnetic beads	repulsive magnetic field	2	—	10 pM	1× PBS	[174]
diamond	HIV-1 Tat protein	aptamer	8.3 M urea	3	<15%	1 nM	1 mM PBS	[168]
Gr-Nafion	IFN-γ protein	aptamer	ethanol	80	<8.3%	0.74 pM	human sweat	[171]
Gr-hydrogel	glucose	glucose oxidase	removing hydrogel	10	<5%	200 nM	1× PBS	[175]
graphene	Cu^2+^	L-phenylalanine	0.1 M HCl	3	—	0.17 pM	—	[167]
rGO	dopamine	aptamer	grinding	100	0.15%	370 pM	0.1× PBS	[176]
rGO	DNA	PNA	8.3 M urea	3	8.3%	0.1 pM	1× PBS	[169]

## 8. Guidelines for reporting bioFETs with clinical results

### 8.1. Reporting receiver operating characteristic (ROC) curves

The ROC curve of a biosensor plots true positive rate (sensitivity) versus false positive rate (1−specificity) for various thresholds [177]. The area under the ROC curve (AUC) provides a single metric to evaluate the diagnostic accuracy of a biosensor [178]. A higher AUC indicates better discrimination between positive and negative samples. An ideal biosensor achieves an AUC of 1 while a random guess has an AUC of 0.5. As a rule of thumb, AUC > 0.8 suggests a reliable diagnostic test [179]. Notably, the state-of-the-art bioFETs have achieved an AUC of ∼1 for the detection of SARS-COV-2 viruses in clinical testing due to their ultrahigh sensitivity [50, 143].

The ROC curve offers a standardized approach to compare the performance across biosensors and hence is important in assessing the performance of biosensors in new diagnostic tests [178]. Additionally, it helps determine the optimal threshold for a biosensor, based on the desired balance between sensitivity and specificity for clinical purposes [180]. Thus, when testing clinical samples with bioFETs, it could be important to report the ROC curve.

### 8.2. Establishing proportional bias to gold standard measurements

There are two types of systematic errors that may arise in biosensor measurements: fixed and proportional bias. Fixed bias refers to biosensor readings deviating from true values by a consistent amount, whereas proportional bias entails biosensor readings deviating from true values by a consistent percentage.

Establishing proportional bias between biosensor measurements and gold standard measurements is important in assessing the accuracy and reliability of the biosensor. If a biosensor exhibits notable proportional bias, it may not be suitable for direct substitution with the gold standard. By comprehending the degree and direction of proportional bias, users can make well-informed decisions and take appropriate actions to enhance the biosensor’s performance. Linear regression analysis is a commonly utilized method to identify and distinguish fixed and proportional bias between the biosensor and gold standard measurements [181].

## 9. Summary and outlook

### 9.1. Major takeaways

In summary, significant progress has been made in field-effect biosensing over the past two decades, with the limit of detection advancing from the picomolar range to the attomolar range. The current state-of-the-art bioFETs have achieved an impressive LoD of 1 copy per 100 *μ*l (17 zM) in 1× PBS [8–10, 47], and 15–30 copies per 100 *μ*l (0.25–0.5 aM) in serum [8, 9] for the detection of nucleic acids and proteins. Some of these ultrasensitive bioFETs with sub-attomolar LoD also had a detection time of ten minutes or less [8, 35], meeting the demand for POC.

Such progress has been achieved by enhancing mass transport, biorecognition and binding, and electrostatic control, and minimizing background noise. 2D materials based bioFETs have achieved lower limit of detections than 1D materials, owing to their superior electrostatic control with an atomically thin body and a millimeter-sized channel for enhanced analyte capture.

The Debye limit is overcome by increasing the Debye length with concave surfaces, or nanoporous PEG/PEM coating, or disrupting the formation of the electric double layer by using high-frequency (>MHz) perturbation. Alternatively, analytes can be brought within the electric double layer to surpass the Debye limit.

In terms of parallelization, planar silicon has been used to achieve a packing density of over one million bioFETs per chip, while the packing density of CNT, SiNW, and graphene has reached 10 000, 1000, and 256 bioFETs per chip, respectively. Parallelization can improve the sensitivity, reliability, multiplexing, and throughput of detection.

Additionally, reusability of bioFETs could help to lower cost even further or to enable new applications in real time physiological or biological sensing. BioFETs with reversible binding can be regenerated by a buffer, DI water rinse, or a mild electrolyte bath. For those with strong binding, sensors can be recycled by pH treatment, detergent, or a strong electrolyte bath. The LoD of reusable bioFETs is still limited to 0.1–1 pM, compared to sub-aM LoD benchmark.

### 9.2. Current status of commercialization

Over the past few decades, ISFET based pH sensors have been successfully commercialized since Bergveld reported the invention of the ISFET in 1970 [182]. Today, many companies such as Thermo Fisher Scientific, Sentron, Microsens SA, and Honeywell offer commercial handheld ISFET pH sensors. Additionally, Ion Torrent [183] (now a division of Thermo Fisher Scientific) and DNA Electronics [184] have employed massively parallelized ISFET arrays for next-generation sequencing by detecting pH changes during DNA/RNA synthesis.

Recently, many companies around the world are attempting to develop commercial FET biosensor products for intrinsic molecular charge detection (e.g. charge of DNA, proteins, and small biomolecules). For instance, Molsentech has launched a COVID-19 testing platform using FET biochips that deliver results in a few minutes with accuracy comparable to PCR tests [185]. IMEC has presented finFET-based biosensors for high-sensitivity molecule detection [186, 187]. Helios Bioelectronics is developing bioFETs for cancer biomarker detection, miRNA profiling for neurodegenerative diseases, and rapid diagnosis for sepsis [188]. Grapheal is making graphene bioFETs for continuous wound care monitoring and *in vitro* diagnostics [189].

### 9.3. Barriers to translation

Despite significant progress in literature in enhancing sensitivity, parallelization, and reusability of field-effect biosensors, there remains a significant gap between academic research and practical point-of-use applications beyond pH sensing. We discuss here the gaps and major barriers, in our opinion, to translate FET biosensors from the lab to the marketplace.

• Reliability. A reliable biosensor should maintain its performance over time (stability) and produce similar results when the same sample is tested multiple times with the same biosensor (repeatability) and with different biosensors (reproducibility). Reliability is crucial in biosensing as it directly impacts diagnostic accuracy. One of the major challenges with field-effect biosensors is their stability. The immobilization of bio-receptors on the sensor surface can degrade over time [190], leading to reduced sensitivity and reliability. Additionally, un-passivated semiconductor channels made from nanomaterials can also degrade in biological fluids. For example, MoS_2_ undergoes slow hydrolysis in aqueous solutions [191]. Furthermore, SiO_2_ is a commonly used dielectric but it is less than ideal for biosensing, as there could be charge traps that accumulate over time in salt solution [192]. Coatings SiO_2_ surfaces with SiN_*x*_ can block the passage of ions [193]. Stoichiometry plays a crucial role in determining the stability and lifetime of SiN_x_; SiN*_x_* with a higher silicon content exhibits reduced stability when exposed to a salt solution [194, 195]. In comparison, high-k dielectrics such as Al_2_O_3_ and HfO_2_ are much more durable in salt solutions, leading to reduced measurement drift, leakage, and noise of bioFETs [196, 197]. Repeatability and reproducibility depend on minimizing variability in sensor fabrication, surface functionalization, and measurement protocols. To increase reliability even for the sensing of a few analytes, bioFETs arrays are needed to provide redundancy and should use negative controls to compensate for sensor drift. For example, a practical design would include a small panel with 5–20 markers, each having hundreds of sensors within the same chip. This approach would increase reliability and provide robustness to the biosensing process.
• Direct interface to biological fluids. Biological fluids may contain various interfering components, such as cells, proteins, or enzymes, that can affect the accuracy and reliability of the assay or even damage the sensing surface of the device. Antifouling measures are typically necessary for sensors to function effectively over time with unprocessed biological fluids [198–200]. Blood analysis has traditionally been the gold standard for diagnostics, as blood is perhaps the most information-rich biological fluid in the body [200]. However, blood-based devices need some form of sample processing before analysis. Sample processing may involve clotting, centrifugation, and filtration to remove unwanted species, inactivation of inhibitors, etc as well as sample dilution to optimize the concentration of target molecules. Saliva and sweat have a lower potential for biofouling compared to blood, due to their simpler constituents and lower concentrations of biomolecules [201, 202]. Notably, unprocessed saliva testing has been achieved using aptamer-based graphene FET biosensors for detecting SARS-COV-2 at levels as low as 7 to 10 viruses [203]. Furthermore, a wearable aptamer-based In_2_O_3_ thin-film FET biosensing system has been developed for noninvasive cortisol monitoring [151].
• Sensor preparation time. Except for direct pH sensing, every sensor needs preparation (functionalization) before actual use, in which a capture agent is attached to the sensor surface. Generally, it takes several hours to days to functionalize sensor surfaces with bioreceptors [8, 9, 25, 31–33, 35, 49, 58, 59, 96–98, 204]. For instance, while a type of graphene bioFETs could detect SARS-COV-2 cDNA in artificial saliva down to 17 zM in 6.5 min, it takes 14.5 h to functionalize the graphene FETs before use [8]. Since ELISA plates and DNA microarrays have been commercialized, which take at least a few hours to functionalize, bioFET sensor surfaces can also be functionalized with antibodies, aptamers, and DNA probes within acceptable preparation time.
• Reusability. For continuous monitoring of analyte concentration *in vivo*, from skin, or for analysis of *ex vivo* tissues in real time, bioFETs need to be reversible or regenerative, miniaturized, multiplexed, energy-efficient, and integrated with data processing and wireless transmission units. At the cost of LoD, receptors can be engineered to exhibit fast binding kinetics, facilitating reversible binding for continuous biosensing [205]. Alternatively, real-time monitoring can be accomplished by regenerating the biorecognition layer following each measurement. Ideal regeneration for process monitoring would remove the captured target *in situ* using a chemical-free approach and preserve the biorecognition layer for subsequent measurements [206]. One possible solution is to apply a repelling gate voltage to weaken the electrostatic attraction between the probe and the analyte for fast desorption [150].

### 9.4. Commercial viability

We assess here the commercial viability of field-effect biosensors by considering cost implications and comparative advantages, assuming successful resolution of all technical impediments to translation.

If the FET biosensor is for one-time use, then its cost must be low enough to justify the use of the sensor in that application. For sequencing applications, users are willing to pay higher costs ⪆ $1000 per genome, hence justifying the cost of one large array of FET sensors per assay. For personalized diagnostics, the cost per test must be low ∼$10–$100 or less, depending on reimbursement costs and the value of the test. For example, for point of care PCR or LAMP tests for COVID-19, the kits are on the order of a few $100. For additional context, recent COVID-19 antigen tests typically cost $10–$30 per kit, and home pregnancy tests generally run anywhere from $8 to $15.

To estimate the fabrication cost of large FET sensor arrays, we take a baseline number of $10 per square centimeter for 180 nm CMOS lithography performed at a foundry at production scale [207]. At such technology nodes, over 13 million FET sensor array units can be integrated on a chip with a size of 15 mm × 15 mm [18]. This capacity is more than sufficient to host tens of biomarkers, with hundreds of FET biosensors allocated to each biomarker. Therefore, the cost of one large array of FET sensors per assay below ∼$10 is justified if they can be manufactured at production scale.

Electrochemical biosensors stand as formidable contenders to FET biosensors, harnessing the full array of benefits offered by electrical chips for point-of-care applications. Moreover, electrochemical biosensors offer a cost advantage over FET biosensors due to their simpler design and easier fabrication.

However, the decisive edge that FET biosensors possess over their electrochemical counterparts lies in their capability of ultralow limit of detection. In practice, electrochemical biosensors typically operate within LoD ranges of the micromolar to nanomolar levels, with potential extensions into the picomolar to femtomolar range [3, 208, 209]. Meanwhile, FET biosensors could achieve significantly lower LoD. For instance, an organic electrochemical biosensor using field-effect transduction achieved a LoD of 10 pM for the detection of ATP, which is four orders of magnitude lower than the LoD (106 nM) achieved when using electrochemical transduction for the same sensor [210]. This stark contrast underscores the potential of FET biosensors to seamlessly complement electrochemical sensors in contexts demanding a lower LoD.

While electrochemical sensing has demonstrated its utility in wearable sweat sensors for detecting ions and small molecules [211–213], the realm of detecting DNA, proteins, and larger biomolecules in sweat remains a challenge due to their exceedingly low concentrations [214, 215]. Herein lies an area where FET biosensors may assert a distinct advantage over their electrochemical counterparts.

Furthermore, as our comprehension of the intricate interplay between biochemistry and physiology advances, the potential to establish nuanced links between trace levels of biomarkers and overall health becomes increasingly viable. This evolving understanding paves the way for the strategic deployment of FET biosensors in the early detection of diseases, capitalizing on biomarkers present at the femtomolar to attomolar levels.

### 9.5. Considerations around portability

Traditional FET biosensor setups typically rely on complex and bulky measurement instruments for data acquisition and processing, such as off-the-shelf semiconductor parameter analyzers. These systems are often nonportable and hard to use. For true point-of-care use, it is important to develop low-power, miniaturized analyzers that can operate on battery or energy harvesting systems [216, 217]. For instance, 300 CNT FET sensors were integrated with CMOS electronics on a chip using 0.25 *μ*m very-large-scale-integration technology for sensor control, calibration, and signal processing, with a total power consumption of 62.5 *μ*W [218]. Moreover, a portable CNT-FET based COVID-19 testing system was developed with a size of 12.45 cm × 14.9 cm × 10.4 cm. It consists of a raspberry Pi, two 16-bit digital to analog to converters (DACs), a 24-bit analog to digital converter (ADC), a biosensor array, a trans-impedance amplifier (TIA), and low-pass filters [219]. The final test results could be read directly from the display screen of the test instrument.

The widespread availability of smartphones with powerful processing capabilities offers an excellent platform for point-of-care analyzers. Researchers are exploring ways to leverage smartphones for data acquisition, analysis, and wireless communication, making the systems more portable and user-friendly [220]. In a recent study, a FET biosensor array for urine analysis was integrated with a device control panel for data acquisition, conversion, and transmission, and a smartphone was used for data analysis and display [147]. The overall dimension of this portable integrated system was 15.2 cm × 6.5 cm × 2.4 cm. The operation processes are as follows: the micro-controller unit (MCU) in the data conversion module reads instructions from the smartphone via a wireless Bluetooth unit and transfers the instructions to voltage signal through a DAC. The voltage following module then amplifies the voltage signal and applies voltage toward the FET biosensor array. Subsequently, the MCU sequentially accesses the measured current signal from the FET biosensor array and converts the signal into readable information by an 8-to-1 multiplexer, a TIA, and an ADC. The Bluetooth unit exports the readable information to a smartphone. The application program, which incorporates neural network algorithm and a diagnosis interface, analyzes the received information to display the diagnosis results.

In the era of the internet of things, sensors can operate autonomously without relying on other devices (e.g. PC, tablets, or smartphones) [221]. The developed hardware can be integrated into a cloud-based platform, leveraging the computational power of the cloud to perform innovative algorithms for calibration. Results and configurations can be accessed through a web page without the need to install dedicated application programs or software.

## Data Availability

All data that support the findings of this study are included within the article.

## Conflicts of interest

RB discloses advisory role in Helios Bioelectronics, and financial interests as co-founder in Prenosis, and LabSimply. SC discloses no conflicts of interest.
